# Tonic exploration governs both flexibility and lapses

**DOI:** 10.1371/journal.pcbi.1007475

**Published:** 2019-11-08

**Authors:** R. Becket Ebitz, Brianna J. Sleezer, Hank P. Jedema, Charles W. Bradberry, Benjamin Y. Hayden

**Affiliations:** 1 Department of Neuroscience and Center for Magnetic Resonance Research University of Minnesota, Minneapolis, MN, United States of America; 2 Department of Neurobiology and Behavior, Cornell University, Ithaca, NY, United States of America; 3 NIDA Intramural Research Program, National Institute on Drug Abuse, Baltimore, MD, United States of America; Harvard University, UNITED STATES

## Abstract

In many cognitive tasks, lapses (spontaneous errors) are tacitly dismissed as the result of nuisance processes like sensorimotor noise, fatigue, or disengagement. However, some lapses could also be caused by exploratory noise: randomness in behavior that facilitates learning in changing environments. If so, then strategic processes would need only up-regulate (rather than generate) exploration to adapt to a changing environment. This view predicts that more frequent lapses should be associated with greater flexibility because these behaviors share a common cause. Here, we report that when rhesus macaques performed a set-shifting task, lapse rates were negatively correlated with perseverative error frequency across sessions, consistent with a common basis in exploration. The results could not be explained by local failures to learn. Furthermore, chronic exposure to cocaine, which is known to impair cognitive flexibility, did increase perseverative errors, but, surprisingly, also improved overall set-shifting task performance by reducing lapse rates. We reconcile these results with a state-switching model in which cocaine decreases exploration by deepening attractor basins corresponding to rule states. These results support the idea that exploratory noise contributes to lapses, affecting rule-based decision-making even when it has no strategic value, and suggest that one key mechanism for regulating exploration may be the depth of rule states.

## Author summary

Why do we make mistakes? We seem to have the capacity to identify the best course of action, but we do not always choose it. Here, we report that at least some mistakes are due to exploration—a type of decision-making that is focused on discovery and learning, rather than on choosing the best option. This is surprising because many views of exploration assume that exploration only happens phasically—when the circumstances suggest that you should abandon your previous course of action and make a new plan. However, here, we find evidence that exploration drives decisions to change your behavior both when change is helpful and when it is a mistake. More work is needed to understand why we explore tonically, but it is possible that tonic exploration may been so useful over evolutionary time that our brains evolved to continue to explore today, even when it has no strategic benefit in the moment. For example, a tonic algorithm for exploration could reduce the effort required to make decisions or prepare us to take advantage of unexpected opportunities.

## Introduction

Decision-makers can implement arbitrary rules (i.e. stimulus-response mappings) and flexibly change them when contingencies change [1,2]. Yet even sophisticated decision-makers occasionally fail to implement well-learned rules. Why do these lapses occur? In the past, lapses of rule adherence have been tacitly dismissed as the product of ancillary nuisance processes, such as memory deficits, sensorimotor noise, or disengagement [3–6]. An alternative view is that some lapses occur because of the same adaptive processes that allow rule-learning and cognitive flexibility in a changing environment. That is, lapses may be caused, in part, by exploration.

In changing environments, decision-makers balance the exploitation of valuable strategies with exploration. That is, they occasionally deviate from previous rules in order to sample alternative options and learn about the environment [7–12]. In some algorithms for exploration, the decision to explore is gated by uncertainty about the correct action [9,11,13]. We will call these phasic exploration algorithms, because exploration only occurs when reducing perseveration has the greatest benefit. Conversely, in what we will call tonic exploration algorithms, the decision to explore does not entirely depend on the value of exploration, but instead also occurs spontaneously—even when there is no benefit to exploration [9,11]. Although tonic exploration may appear suboptimal, exploring tonically eliminates the need to calculate the value of exploration at every time step, is robust to errors in calculating the value of exploration, and it can perform nearly as well as phasic exploration in many circumstances [8,11,14]. However, tonic exploration also has costs: when the environment is stable, it will produce errors of rule adherence that have no immediate strategic benefit. That is, it would cause lapses.

It is not clear whether lapses of rule adherence are due to the same exploratory processes that underlie our capacity for flexibility. If so, this could provide novel insights into both exploration and into disorders in which lapse rates are abnormal (e.g. [15–17]). Perhaps the best way to address this question is by looking at behavior in a task that has both stable periods—in which there is no uncertainty and exploratory noise has no strategic benefit—but also rapid changes in reward contingencies that require adaptation and learning. That is, in an extreme example of the change-point tasks used to study adaptation to volatility in reward contingencies [18–21]. If tonic exploration causes both lapses and flexibility, then spontaneous lapses during stable periods should predict the ability to discard a rule when the environment does change. That is, lapse rates should be negatively correlated with perseverative errors. An alternative hypothesis is that exploration is phasic, generated only at change points. If so, then lapse rates would not be correlated with perseverative errors (because they are caused by different processes), or perhaps positively correlated (because they are both errors of task performance).

Furthermore, if lapse rates and adaptation at change points are both caused by tonic exploration, then it should be possible to simultaneously regulate both behaviors via perturbing the underlying common cause. One candidate perturbation is chronic cocaine exposure, which has long been known to reduce cognitive flexibility, though the nature of these effects is complex [22–26]. For example, cocaine abusers make more perseverative errors in classic rule-shifting tasks such as the Wisconsin Card Sort Task (WCST, [27–30] and both rodents and monkeys exposed to cocaine show deficits in reversal learning [31,32], failing to change behavior in the face of aversive outcomes [33]. This striking inflexibility may even contribute to the cycle of abuse in cocaine users [23,26,34]. However, although there is convincing evidence that chronic cocaine exposure causes inflexibility, these effects have defied simple explanation in terms of changes in common behavioral parameters such as reward processing or learning rates.

If chronic cocaine abuse increases inflexibility via decreasing tonic exploration, then it should not only cause perseverative errors, but also decrease lapse rates. It could, for example, simultaneously decrease flexibility yet improve performance in set-shifting tasks. Indeed, at least one observational study reported that human cocaine abusers performed better in the WCST, compared to controls [35]. However, it remains unclear whether chronic cocaine can causally simultaneously reduce lapse rates and increase perseverative errors within the same subjects. Addressing this question has the potential to reconcile seemingly paradoxical results in the cocaine literature, and, at the same time, to address a fundamental question about whether lapses are caused by the same tonic exploration process that facilitates adaptation and learning.

Here, we examined behavior of rhesus macaques performing the cognitive set shifting task (CSST, [36–40], a primate analogue of the WCST, both before and after exposure to cocaine. This task is ideal to address the present question because it combines a change point task with a rule-based decision-making task that requires monkeys to repeatedly apply a cognitive rule. Consistent with tonic exploration, we found evidence of a common cause of lapse rates during stable periods and flexibility following change points. Cocaine not only reduced flexibility, but simultaneously and proportionally decreased lapse rates, suggesting that cocaine regulates tonic exploration. Finally, we fit a model to the dynamics of behavior, in which cocaine decreased exploration via deepening the attractor basins that correspond to rule states. Together these results suggest that exploration occurs tonically and may be well-described as variation in the depth of attractor basins corresponding to rule states.

## Results

**Fig 1:**
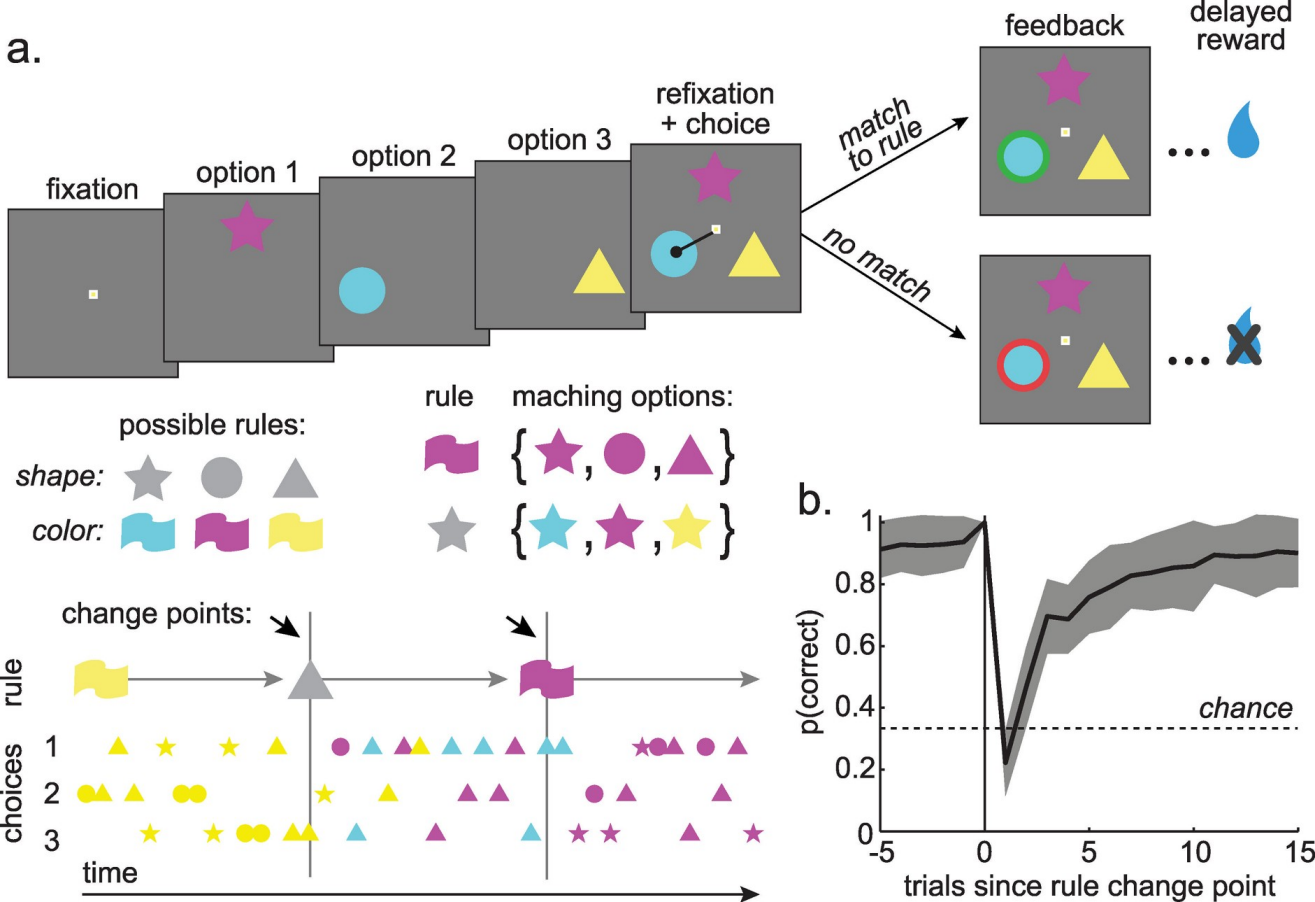
Task design and baseline behavior. A) The CCST task. Three options, which differed in both shape and color were sequentially presented. Choosing an option that matched the rewarded rule produced a green outline around the chosen option and a reward. Choosing either of the other two options produced a red outline and no reward. Middle row, left: Rules could be any of the three shapes or any of the three colors. Right: The options that matched a rule were the set of stimuli that shared the rule’s feature. Bottom: After the monkeys achieved 15 correct choices, the rewarded rule changed, which forced the monkeys to search for the new rule. B) Percent correct as a function of trials before and after rule changes. The 0^th^ trial is the last trial before the rule changed. Gray shading +/- STD.

Two macaques performed 147 sessions of a primate analogue of the WCST (the CSST [36–40]; **Fig 1A**) before and after chronic self-administration of cocaine (n = 89 baseline sessions before cocaine administration, monkey B: n = 62, monkey C: n = 27; n = 58 post-cocaine sessions after, monkey B: 33, monkey C: 25). On each trial monkeys were sequentially offered three choice options that differed in both color and shape (drawn from nine possible combinations of three colors and three shapes). One of the six stimulus features was associated with reward. The rewarded feature (i.e. rule) was chosen randomly and remained fixed until a rule change was triggered (by 15 correct trials under this rule). Rule changes were not cued.

**Fig 2:**
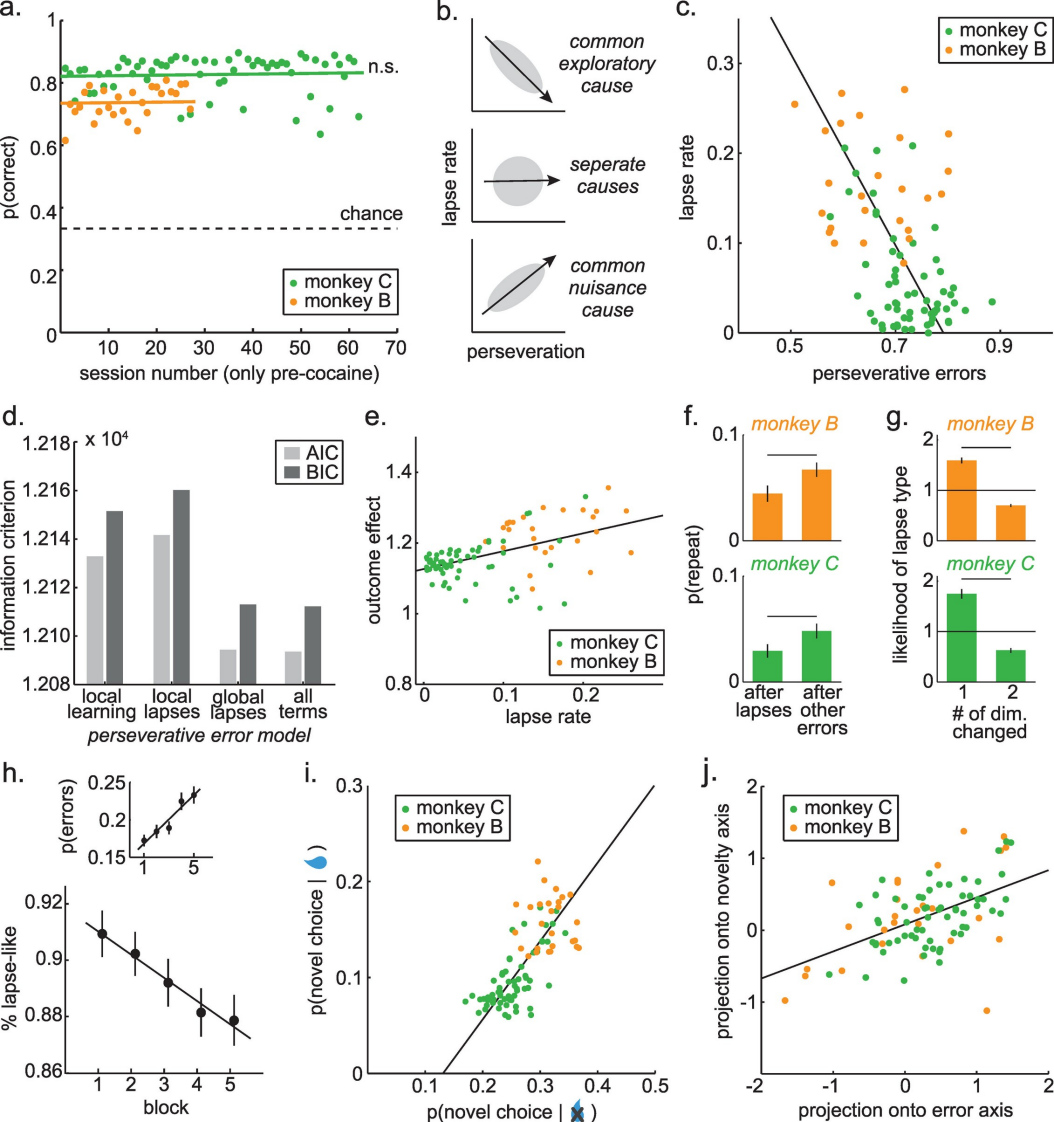
Behavior in baseline sessions. A) Percent correct as a function of session-number in the baseline sessions, plotted separately for monkey C (green dots) and monkey B (orange). Lines = GLM fits (Results). n.s. = not significant. B) Possible relationships between lapse rates and perseverative errors under different hypotheses. Top) A negative correlation if some spontaneous lapses are caused by the same exploratory process that facilitates learning and reduces perseveration at change points. Middle) No correlation if lapses and perseveration are caused by different underlying error processes. Bottom) A positive correlation if lapses and perseveration are both caused by a common error process, such as task disengagement or a failure to learn the reward contingencies. C) The observed relationship between lapses in the 10 trials proceeding change points and perseverative errors in the 5 trials after change points. D) Model comparison asking whether perseverative errors are more closely related to the rate of learning or lapse rate in the last block or to the global lapse rate in that session. E) The correlation between lapse rates and the outcome effect index, a whole-session measure of learning rate. F) The probability of repeating a choice made in error during lapses, compared to other errors in monkey B (top) or monkey C (bottom). G) The frequency that lapses deviate from the last choice in either 1 or 2 stimulus dimensions, normalized by the expected frequency of that choice. H) Changes in errors over the course of each session. Sessions are divided into five equal blocks. Top) Total probability of errors by block. Bottom) proportion of errors that were lapse-like by block. I) The correlation between the likelihood of novel choices (matching neither the last color nor last shape), given reward delivery and omission. J) Relationship between the effects illustrated in panels C (x-axis) and panel K (y-axis). Best fit lines = ordinary least squares. Bars = standard errors.

Monkeys chose the most rewarding option frequently (81.4% of trials ± 6.5% STD across sessions, monkey B = 83.9% ± 5.8% STD, monkey C = 77.1% ± 5.7% STD; average of 576 trials per session, 470 rewarded) and adapted quickly to rule changes (**Fig 1B**). Most errors were perseverative (repeated either the color or shape of the previous option; 64 ± 8.5% STD across sessions; average of). Pre-cocaine sessions were collected after 3 months of training. We observed no measurable trend in performance across the pre-cocaine sessions (**Fig 2A**; percent correct, GLM with terms for main effects of monkey and session number, session number beta = 0.0002, p = 0.6, df = 86, n = 89). Thus, performance had reached stable levels before data collection began.

### Lapse rates and perseverative errors are negatively correlated

Lapses and perseverative errors could be related (or unrelated) for a variety of reasons (**Fig 2B**). First, if lapses are caused by the same process that helps to discard a rule when it is no longer rewarded (e.g. tonic exploratory noise) then lapse rates would be negatively correlated with perseverative errors across sessions. Second, if lapses and perseverative errors are regulated by different processes (e.g. if lapses occur because of a transient memory deficit, while perseverative errors occur because of a failure of inhibitory control), then the frequency of lapses and perseverative errors would not be correlated. Third, if some nuisance process causes both types of errors, then lapses and perseverative errors might even be positively correlated. For example, fatigued or disengaged animals might learn more slowly, taking longer to discover rules and making more lapses before a rule switch. But, at the same time, slowed learning would increase the time necessary to discard a rule once it has been learned, leading to more perseverative errors after a rule switch.

We compared relative frequency of perseverative errors in the five trials after change points (when learning was maximal; **Fig 1B**) with lapse rates in the ten trials before change points (a non-overlapping subset of trials in which learning had reached asymptote). Lapse rates and perseverative errors were negatively correlated (**Fig 2C**; both monkeys: Pearson’s r = -0.52, p < 0.0001, n = 89). This was not a trivial consequence of a performance offset between the monkeys: the effect was strongly significant within the monkey in which we had more baseline data (monkey C: n = 62 sessions, r = -0.45, p < 0.0002; same sign in monkey B: n = 27 sessions, r = -0.26, p = 0.25). There was also no evidence that the effect magnitude changed over time with experience in this monkey (monkey C: sessions < 20: 19 sessions, r = -0.49, p < 0.05; sessions 20+: 43 sessions, r = -0.50, p < 0.001) and the effect seemed to go down, if anything, in the monkey in whom we had fewer baseline sessions (monkey B: sessions < 20: 19 sessions, r = -0.34, p = 0.15; sessions 20+: 8 sessions, r = 0.14, p = 0.74; though it increased again in the post-cocaine sessions: n = 33, r = -0.37, p < 0.05). This negative correlation was apparent regardless of whether we examined lapses where choices changed in both dimensions (both monkeys: Pearson’s r = -0.60, p < 0.0001) or lapses that differed in only one dimension (both monkeys: Pearson’s r = -0.38, p < 0.0002). There was no increase in lapses in anticipation of change points, suggesting that this effect was due to an offset in the rate of lapses throughout the stable period not to the monkeys’ attempts to time change points (**S1 Fig**). Thus, the negative correlation between lapses and perseverative errors indicates that the rate of lapses in rule adherence is positively correlated with the ability to discard a rule when it is no longer rewarded.

Lapse rates in one epoch cannot directly cause flexibility in another epoch (or vice versa), so this correlation implies that both behaviors share some common, underlying cause. One possibility is tonic exploration, which would cause monkeys to occasionally sample an alternative to the current best option, regardless of change points. Another possibility is that monkeys may simply fail to learn in some subset of blocks, which would cause lapses (because the rule is never discovered) and reduce perseverative errors (because a rule that is never discovered is cannot persevere). The failure-to-learn view predicts that perseverative errors in one block should be best explained by the lapses in the immediately preceding block. However, the probability of perseverative errors in each individual block was best explained by the global lapse rate for the session, not by the lapse rate or the rate of learning in the previous block (**Fig 2D**; see Methods; last-block lapse rate model: log likelihood = -6063.4, AIC = 12133, BIC = 12152; last-block learning rate model: log likelihood = -6067.8, AIC = 12142, BIC = 12160; global lapse rate model: log likelihood = -6044.2, AIC = 12094, BIC = 12113; best model = global lapse rate model, all other AIC and BIC weights < 0.0001). Thus, the negative correlation between lapse rates and perseverative errors was not due to a failure to learn in some blocks, but instead to some global common cause, such as tonic exploration.

### Lapses are best explained by exploration, not fatigue or disengagement

If lapses are just due to a nuisance process like disengagement or fatigue—and the negative correlation between lapses and perseverative errors were due to some trivial variability in learning across sessions—then the sessions with the highest lapse rates should be the ones with the lowest learning rates. On the other hand, the purpose of exploration is to learn about the environment [11] and previous empirical studies report that learning is enhanced during exploration [8]. Therefore, if some lapses are caused by tonic exploration, then the sessions with the highest lapse rates should also be the ones with the highest learning rates. Indeed, the sessions with the highest lapse rates were the ones with the most learning. Across monkeys in the baseline sessions, lapse rates were positively correlated with the effect of reward outcomes on the decision made on the next trial (the “outcome effect index”; see Methods) (**Fig 2E;** both monkeys: Pearson’s r = 0.49, p < 0.0001, n = 89 pre-cocaine sessions). The positive correlation was apparent within each monkey in the full dataset (monkey B: r = 0.66, p < 0.0001, n = 60; monkey C: r = 0.39, p < 0.0002, n = 87; both together: Pearson’s r = 0.44, p < 0.0001, n = 147). This means that learning was highest on sessions when lapse rates were highest, consistent with the idea that lapses were due to an underlying drive to learn, rather than disengagement with the task or trivial variability in learning rates across sessions.

Next, we asked whether learning was enhanced during lapses themselves, compared to other errors. To the extent that the monkeys are learning from errors, they will avoid choices made in error. If learning is increased during lapses, we reasoned that monkeys should be less likely to repeat lapse choices, compared to other choices made in error. Indeed monkeys were more likely to avoid repeating lapse choices in the next two trials (**Fig 2F**; mean decrease in probability of repetition after lapses compared to other errors = -0.02, 95% CI = [-0.01, -0.03], p < 0.0001, t(88) = -4.67, paired t-test; monkey B: effect size = -0.02, p < 0.005, t(26) = -4.62; monkey C: effect size = -0.02, p < 0.001, t(61) = -3.53; similar results for 3 or 5 trials into the future). This was an artifact of some greater tendency to repeat rewarded choices in the vicinity of lapse errors because there was no change in the probability of repeating rewarded choices (± 1 choice from a lapse or other error, effect size = -0.005, p > 0.6). These results suggest that learning was enhanced during lapses, consistent with an underlying exploratory cause of lapses.

Together, these results suggest that at least some errors of rule adherence are due to tonic exploratory noise, rather than to nuisance processes. However, it is important to note that tonic exploratory noise implies that the timing of exploration is random, not the choice of what to explore. Lapses may still target important or valuable options, meaning they could reflect a tonic, but directed form of exploration [12]. This would lend further support to the idea that lapses are not solely caused by nuisance processes. In this task, monkeys can lapse by choosing options that differ in both dimensions from the previous choice or options that differ in only one dimension. During stable periods, changing both dimensions would never produce rewards and would provide less information about which choice feature caused the last reward (**S2 Fig**). Thus, the smartest strategy would be to preferentially lapse in only one dimension. This is exactly what we found (base rate of change 1 dimension lapses = 4.5%, change 2 dimension lapses = 3.9%, difference = 0.006, 95% CI = [0.001, 0.010], p < 0.02, t(88) = 2.49, paired t-test; monkey B: effect size = 0.008, p = 0.19, t(26) = 1.34; monkey C: effect size = 0.005, p < 0.02, t(61) = 2.26). This pattern is more striking when you consider that, by chance, lapses should change 2 dimensions more frequently than 1 (see Methods). Nevertheless, lapses were 1.7 times more likely than chance to change in only 1 dimension (**Fig 2G**; sig. more frequent than chance at 1x, 95% CI = [1.57, 1.84], p < 0.0001, t(88) = 10.29; monkey B: 1.6x, p < 0.0001, t(26) = 10.46; monkey C = 1.75x, p < 0.0001, t(61) = 7.93). Thus, although lapses occurred during periods in which they could not improve task performance, these were still smart, information-seeking choices.

Exploration tends to occur most frequently early in experimental sessions—when learning is most valuable—an observation known as the “horizon effect” [12,41,42]. Therefore, we reasoned that if lapses are caused by exploration, they should occur less frequently as the session progresses. In contrast, nuisance processes like fatigue or disengagement tend to increase over the course of an experimental session, as animals become satiated and bored, so any nuisance cause of lapses would increase their frequency over the course of a session. Indeed, the animals’ tended to make more errors overall as the sessions progressed (**Fig 2H**; GLM predicting error from quantile binned sessions, beta = 0.016, p < 0.0001, n = 147 divided into 5 equal bins; identical results for 10 bins, though there were a large number of empty cells). However, the proportion of these that were lapse-like decreased over the course of the session (**Fig 2H**; beta = -0.008, p < 0.002). This was especially pronounced when we confined our analyses to the stable periods (beta = -0.011, p < 0.02). A decrease in the relative proportion of lapses is exactly what we would expect if one latent cause of lapses—such as a tonic drive to explore—decreased over the course of the session, while a more general cause of errors—such as fatigue or disengagement—tended to increase.

### A common cause of novel choices, regardless of outcome

It remained possible that lapses were negatively correlated with perseverative errors, because of some artifact in how we calculated lapses or perseverative errors. Therefore, we next asked whether there was other behavioral evidence that exploration was tonic, occurring both when it was immediately helpful and when it was not. In this task, the outcome of the previous trial provides perfect information about whether or not that choice was correct. If monkeys were rewarded on the last trial, then either the color or shape of the last choice matched the rewarded rule and the best response is to repeat either the color or shape or both in the next trial. Conversely, if the monkeys were not rewarded, then neither the color or shape of the last choice was consistent with the rewarded rule and the best response is to choose a novel option—one that matches neither the color nor the shape of the previous choice. However, tonic exploration would sometimes cause monkeys to choose novel options following reward delivery—when it is clearly incorrect to do so. Indeed, the monkeys did choose novel options after both reward delivery (monkey B: 15.8% novel choices, monkey C: 9.6%) and omission (monkey B: 31.6% novel choices, monkey C: 25.2%). Tonic exploration not only predicts that these choices should occur, but that their frequency should be governed by a common underlying process. That is, the frequency of novel choices after reward delivery should be correlated with the frequency of novel choices after reward omission. Indeed, these choices were strongly correlated (**Fig 2I**; Pearson’s r = 0.72, p < 0.0001, n = 89). This was individually significant within the animal in which we had more baseline sessions (monkey C: n = 62 sessions, r = 0.68, p < 0.0001; monkey B: n = 27 sessions, r = -0.04, p = 0.9). Thus, the monkeys’ decisions to deviate from choice history—to try something new—also co-varied, regardless of whether or not that was correct, consistent with a common cause.

If the preference for novelty were due to the same underlying exploratory mechanism that caused the negative relationship between lapses and perseverative errors, then we would expect these two effects to be positively correlated across monkeys and sessions. The axis on which each pair of effects endogenously co-varied (i.e. the best fit lines in **Fig 2C and 2I**) reflects the linear portion of all common underlying influences. Any variation in these underlying influences would shift where the data fell along this single dimension. Therefore, to determine whether the preference for novelty was due to the same underlying exploratory mechanism, we projected each pair of effects onto these best fit lines (see Methods) and asked whether variation in the common cause of lapses and perseverative errors predicted variation in the common cause of novel choices. There were strong positive correlations between the two effects in both monkeys individually (**Fig 2J**; monkey C: n = 62 sessions, r = 0.56, p < 0.0001; monkey B: n = 27 sessions, r = 0.50, p < 0.01) and together (Pearson’s r = 0.52, p < 0.0001, n = 89). Thus, the shared tendency to choose novel options, regardless of reward history, was related to the same underlying tonic exploratory process.

### Cocaine self-administration

The baseline behavior suggested that a common, exploratory process regulated the decision to deviate from a rule or choose a novel option, regardless of whether or not it was correct to do so. If this is true, then it should be possible to co-regulate lapses and perseverative errors by regulating this tonic exploratory process process. Therefore, we next allowed both monkeys to self-administer cocaine—exposure to which is known to affect the ability to adapt to a changing environment [22–26,31].

Monkeys self-administered cocaine through an implanted venous port (see Methods). Briefly, for 3 hours each day, 5 days a week, over a total of 6 to 7 weeks (monkey B: 50 days, monkey C: 42 days), monkeys were placed in front of a touch screen display and pressed a centrally located cue a set number of times (see Methods), which resulted in cocaine infusion. Monkeys initially underwent self-administration training (10 days). During this time, the cumulative dose of cocaine self-administered per day increased from 0.8 mg/kg to 4 mg/kg at 3 responses/reward (FR3), followed by a ramp-up period to 30 responses/reward (FR30; 7 days at 4 mg/kg), after which we began examining behavioral data during chronic cocaine exposure. We collected behavior in the morning, while monkeys self-administered cocaine in the afternoon in a separate session (with a minimum of 1 hour of home cage time in between). This experimental design allowed us to determine the long-term effects of chronic cocaine self-administration without the drug “on board” at the time of testing. Over all self-administration sessions, monkey B administered a cumulative total of 179.9 mg/kg of cocaine, while monkey C administered 153.2 mg/kg cocaine.

### Effects of cocaine on behavior

**Fig 3:**
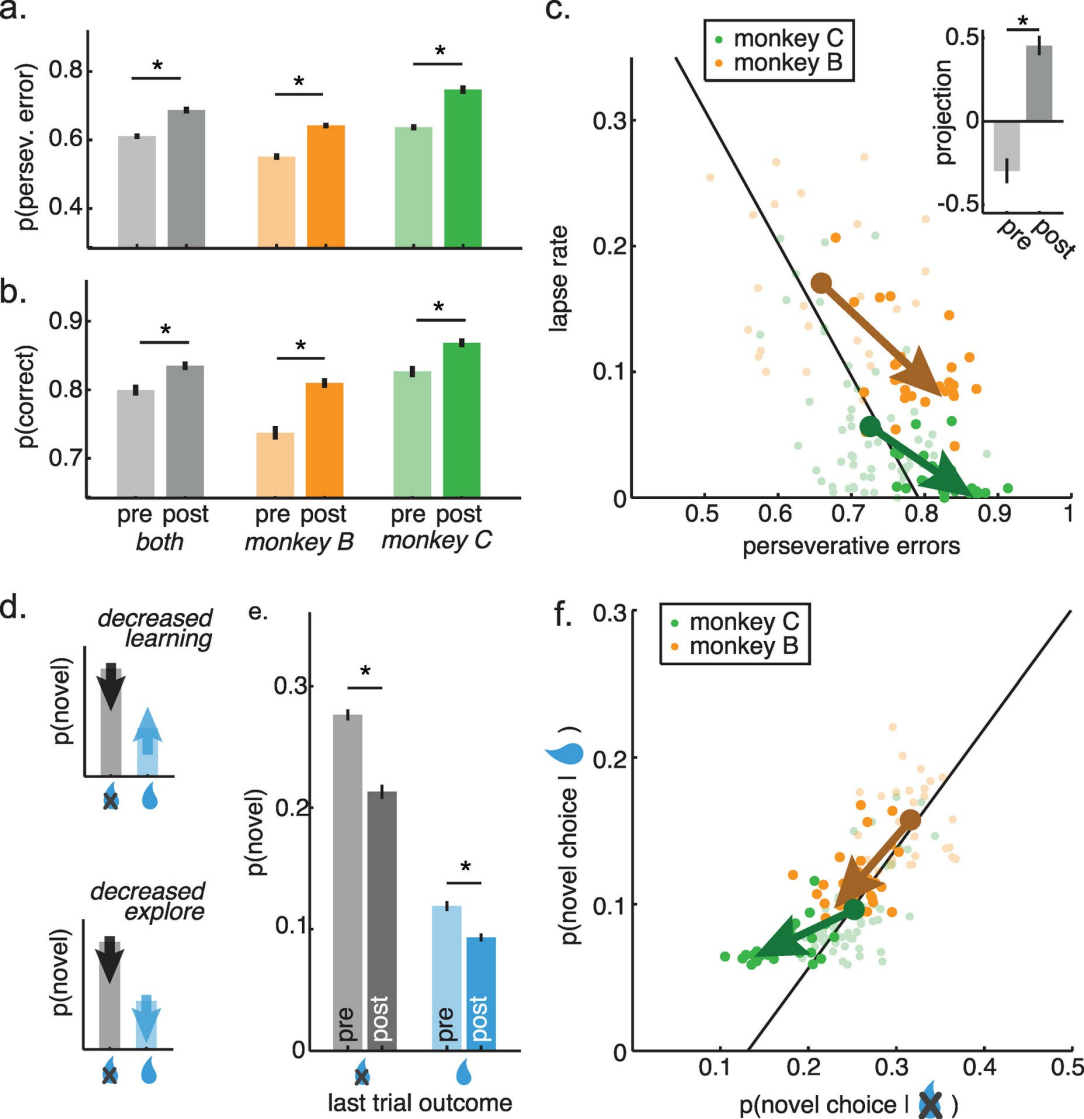
Changes in CSST behavior after cocaine administration. A) The probability of perseverative errors before and after cocaine treatment (before = light, after = dark), plotted together for both monkeys (gray) as well as separately for monkey B (orange bars) and monkey C (green). Error bars +/- SEM throughout and * p < 0.05, two-sample t-test. B) Same as A, for the percent of total correct trials in the pre- and post-cocaine sessions. C) Cocaine’s effects on the relationship between spontaneous lapses and perseverative errors. Same as 2E, but now illustrating post-cocaine sessions (dark) and pre-cocaine sessions (light). The vectors reflect the shift in the mean with cocaine for monkey B (orange) and monkey C (green). E) Cartoons illustrating different hypotheses. Top) If cocaine decreased learning rates, it would reduce effect of past outcomes on future choices, thereby reducing the difference in the probability of novel choices following trials that were or were not rewarded. Bottom) If cocaine decreases exploration, it would reduce all novel choices, without regard to previous reward outcome. E) Change in novel choice probability, plotted separately for reward omission (gray) and delivery (blue). Pre-cocaine = light, post cocaine = dark. F) Cocaine’s effects on the relationship between novel choices after reward delivery (ordinate) and omission (abscissa). Conventions the same as in 3C.

Because chronic cocaine exposure is associated with decreased flexibility and increased perseveration, we first asked whether cocaine administration changed the proportion of perseverative errors. It did (**Fig 3A**; fraction of all errors that were perseverative, post cocaine compared to pre, t-test: p < 0.0001, t(145) = 6.13, mean increase in fraction perseverative errors = 7.7%, 95% CI = 5.1% to 10.0%; monkey B: p < 0.0001, t(58) = 7.70; monkey C: p < 0.0001, t(85) = 6.99). One concern in any study of chronic drug use is that practice alone could change behavior and appear to be a drug effect. To test for this possibility, we developed a generalized linear model (GLM) to differentiate between the effects of drugs and practice (see Methods). There was no effect of practice on perseverative errors (β_2_ = 0.003, p = 0.7) and including a term for session number did not change the magnitude of the effect of cocaine (β_1_ = 0.097, p < 0.0001), indicating that practice explained little, if any, change in perseverative errors in post-cocaine sessions.

If cocaine increased perseveration by decreasing tonic exploration, then it might also improve overall performance in this set-shifting task by reducing lapse rates. Cocaine reduced whole-session error rates (**Fig 3B**; percent correct, post cocaine compared to pre, t-test: p < 0.001, t(145) = 3.36, mean increase = 3.6%, 95% CI = 1.5% to 5.7%; monkey B: p < 0.0001, t(58) = 6.30; monkey C: p < 0.002, t(85) = 3.22). Again, session number did not affect accuracy (β_2_ = 0.001, p = 0.9) and accounting for session number only increased the apparent magnitude of the effect of cocaine (compare 3.6% change to β_1_ = 0.054, p < 0.0005). This was likely driven by the substantial decrease in the frequency of lapses in the 10 trials before change points (Fig 3C; two-sample t-test; monkey B: p < 0.0001, t(58) = 5.57, mean difference = 7.1%, 95% CI = 4.6% to 9.7%; monkey C: p < 0.0006, t(85) = 3.59, mean = 4.0%, 95% CI = 1.8% to 6.2%).

The hypothesis that cocaine regulates a common cause of flexibility and lapses makes a strong prediction: that cocaine should simultaneously shift lapses and perseverative errors along the axis on which they endogenously co-vary (i.e. the best fit line in **Fig 2C**). Therefore, we measured the projection of the pre- and post-cocaine sessions onto the axis along which the two behaviors endogenously co-varied (see Methods). Cocaine significantly shifted behavior along this axis (**Fig 3C**; two-sample t-test, both monkeys: p < 0.0001, t(145) = 7.60, mean shift = 0.77, 95% CI = 0.57 to 0.98). The effect was similar in both monkeys (monkey B: p < 0.0002, t(58) = 4.09, mean = 0.72, 95% CI = 0.37 to 1.07; monkey C: p < 0.0001, t(85) = 5.48, mean = 0.68, 95% CI = 0.44 to 0.93). This result is consistent with the idea that cocaine regulates the underlying cause of both behaviors.

Next, we asked whether cocaine had similar effects on monkeys’ decisions to deviate from their own previous policy. That is, the probability of novel choices (**Fig 2J**). If cocaine decreased learning (i.e. the effect of reward on behavior), then it would decrease the difference in novel choices following reward delivery and reward omission (**Fig 3D, top**). However, if cocaine decreased tonic exploration, then it would instead decrease the probability of novel choices, regardless of reward outcome (**Fig 3D, bottom**). Cocaine decreased the probability of novel choices both after reward omission (when novel choices were the best option, **Fig 3E**; two-sample t-test, both monkeys, p < 0.0001, t(145) = 6.16, mean change = -5.1%, 95% CI = -3.4 to -6.7%; monkey B: p < 0.0001, t(58) = 7.99; monkey C: p < 0.0001, t(85) = 8.57; not due to practice β_1_ = -0.057, p < 0.0001; β_2_ = -0.008, p = 0.1) and after reward delivery (when novel choices were the worst option, both monkeys, p < 0.006, t(145) = 2.83, mean change = -1.7%, 95% CI = -0.5 to -2.9%; monkey B: p < 0.0001, t(58) = 6.97; monkey C: p < 0.001, t(85) = 3.50; not due to practice β_1_ = -0.024, p < 0.002; β_2_ = -0.005, p = 0.2). Thus, cocaine decreased the probability of novel choices, regardless of reward outcome, consistent with tonic exploration.

If these effects are due to cocaine’s effects on tonic exploration, then cocaine should simultaneously alter the probability of novel choices regardless of previous outcome. That is, cocaine should shift novel choice probability along the axis of endogenous co-variability between rewarded and non-rewarded trials (line in Fig 2G). It did so (Fig 3D: two-sample t-test, both monkeys, p < 0.0001, t(145) = 5.78, mean change = 0.49, 95% CI = 0.32 to 0.66; monkey B: p < 0.09, t(58) = 1.73; monkey C: p < 0.0001, t(85) = 7.85). Thus, cocaine appeared to regulate the probability of making novel choices directly, rather than modulating the effect of rewards on novel choices. Because tonic exploration would produce novel choices both when they are useful and when they are not, this result is consistent with the idea that chronic cocaine down-regulates tonic exploration.

### Hidden Markov model

We previously developed a method for differentiating exploration and exploitation in sequential decision-making tasks that uses a hidden Markov model (HMM) to characterize the latent goal states underlying behavior [8]. Here, we extend this approach to the CSST task. An HMM models the dynamics of behavior in a generative framework without making assumptions about the cognitive and/or neural computations underlying choice. We chose this modeling framework because our goals were to identify when an animal was exploring and look for cocaine-related changes in the dynamics of exploration. HMMs are commonly used to make inferences about the latent states underlying observations, like the latent exploratory or exploitative goals underlying choices [8,43]. The inference problem has not yet been solved in a mechanistic framework—a modeling approach which links behavioral features to specific cognitive or neural processes—because it has proven difficult to differentiate exploration from errors of reward maximization [8,13,44]. A mechanistic approach would be particularly challenging here because we do not yet fully understand the psychological and/or neural computations underlying choice in this task. This means that it is not clear whether choices differ from the predictions of a value-maximizing model because they are exploratory or because of model misspecification. (Of course, this is an area of active research: several interesting mechanistic approaches to this task have been proposed [45,46] and future work can build on promising advances in multidimensional choice [47,48] and task switching [49,50]). Because an HMM models the dynamics of latent goals as a system of difference equations, it is also especially analytically tractable—with an HMM, we can directly calculate how cocaine changes the energetics exploration. Thus, the HMM framework was ideally suited for our present goal—to infer the latent exploratory states underlying behavior and measure how cocaine affected them.

**Fig 4:**
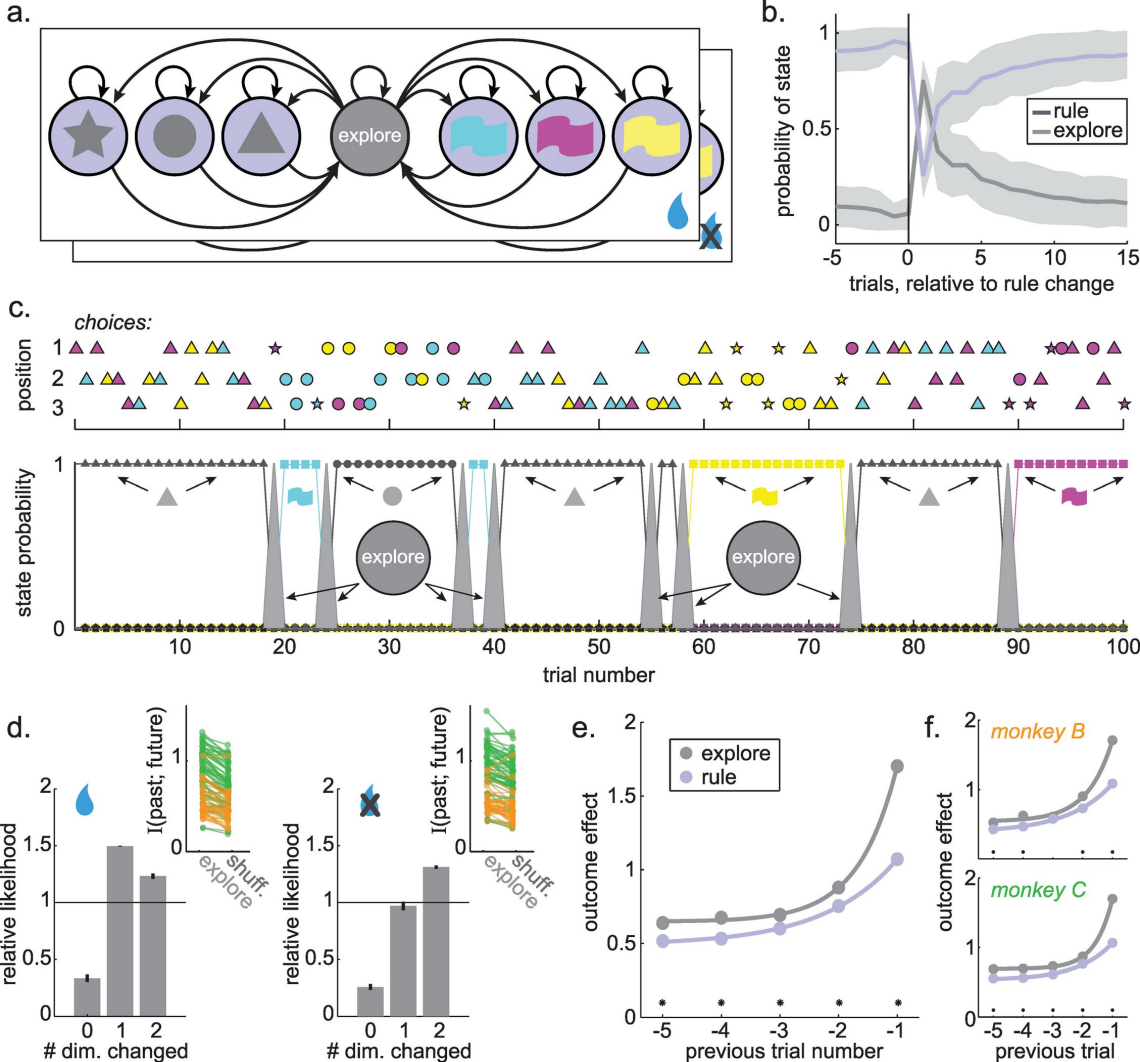
Hidden Markov model (HMM) design and fit to behavior. A) The structure of the HMM, with one latent state for each possible rule, plus one latent “explore state”. Emissions (not shown) match the rule in the rule states, and are randomly allocated during the explore state. The box around the model indicates that this model has multiple “plates”, which depend on the reward of the previous trial (bottom right). Each path (p(transition) between states) depends on whether reward was or was not delivered on the previous trial. B) The posterior probability of explore states and any of the rule states (1-p(explore)) is illustrated as a function of trials relative to change points in the rewarded rule. Shading: +/- STD. C) Example choice sequence and state labels. Top) A sequence of 300 chosen options, separated vertically by whether the chosen option was in location 1, 2, or 3. Bottom) The state probabilities from a fitted HMM. Colored boxes correspond to the color-rule states (blue, yellow, and magenta). Black shapes correspond to shape-rule states (triangle, circle, square). The filled gray line corresponds to the explore state probability. D) Choices made during exploration were organized with respect to choice history. Right) The likelihood that exploratory choices after reward delivery will differ from the previous choice in 0, 1, or 2 stimulus dimensions, normalized by expected frequency of that choice type. Inset) Mutual information for real explore choices compared to explore choices with shuffled choice history for all sessions in monkey B (orange) and monkey C (green). Left) Same as right, for trials following reward omission. E) The outcome effect index for outcomes received during exploration (gray) or during rules (purple), illustrating the effects on 1 to 5 choices into the future. F) Same as E, plotted separately for monkey B (top) and monkey C (bottom).

The HMM we used (**Fig 4A**) assumed that animals were making choices while in one of two discrete types of latent “states”—either they were using a rule, or they were searching for a rule. We term this search state “exploration” for reasons detailed below. Only choices that were consistent with the rule were permitted in rule states, but choices were not constrained during exploration. The model structure was based on two distinct dynamics we found in the behavior (**S2 and S3 Figs**): one associated with repeated choices within a feature dimension (i.e. following a rule) and one associated with rapid samples across feature dimensions with the same half-life as random choices. The discretization of the latent goal states differentiates the HMM from other models, such as a Kalman filter or reinforcement learning models [10,13,51], which would assume some continuous latent state space. However, rules in this task are discrete by design and behavior was well-described by a mixture of discrete states (**S3 Fig)**. To account for the fact that choice dynamics depended on reward (**S5 Fig**), we extended model to allow reward outcomes to affect the probability of transitioning between states (see Methods; [52]). The input-output HMM (see Methods) qualitatively reproduced the reward-dependent state durations we observed in behavior (**S5 Fig**). The performance gradient and optimal combination of model parameters for this task is shown in (**S6 Fig**).

We found that the changes in the latent states inferred by the model (see Methods) were strongly aligned with the change points in the task, indicating that the model was most likely to identify choices as exploratory at precisely the time when the monkeys were actually searching for a new rule (compare **Figs 4B** and **1B**, see **S7 Fig**). For example, the probability of exploration tended to be lower than chance in the 5 trials before change points (sig. decrease in 96% or 85/89 of individual baseline sessions, 2-sided permutation test against 100 label-shuffled datasets; 95% or 139/147 overall). Conversely, the first trial after a change point was more likely than chance to be identified as exploratory in 96% of individual baseline sessions (85/89; 97% or 142/147 overall). An example choice sequence with the associated latent state probabilities is shown in **Fig 4C**.

### Explore-labeled choices were information-maximizing and learning was enhanced

Although similar procedures are used to identify periods of exploration in other tasks [8,43] and explore-labeled choices occurred most frequently when the animals should have been searching for a new rule, it remained unclear whether choices labeled as exploratory here were truly due to exploration. Therefore, we next asked whether explore-labeled choices resembled exploration in other ways. Were these, like lapses, directed choices in which reward learning was enhanced? Indeed, we found that explore-labeled choices were more organized with respect to reward history than we would expect if these were just random choices (**Fig 4D**; sig. higher-than-expected mutual information with the previous choice during explore choices, paired t-test against shuffled control, rewarded on the last trial: 0.23 bits, 95% CI = [0.20, 0.27], p < 0.0001, t(88) = 14.25; not rewarded on the last trial: 0.17 bits, 95% CI = [0.13, 0.20], p < 0.0001, t(88) = 8.79). This was due to two distinct patterns of explore-labeled choices after rewarded and non-rewarded choices. After animals were not rewarded, they were most likely to explore options that differed in both dimension from the previous choice—maximizing the chance of discovering a new rewarded action (**S2 Fig**). Conversely, explore choices after reward tended to differ in only 1 dimension from the previous option—the choice that maximized information about which of the previous two stimulus features produced reward (**S2 Fig**). Thus, exploratory choices were, like lapses, directed to the options that maximized information about which option was best.

Next, we asked whether learning was also enhanced during explore-labeled choices in the baseline sessions. Again, we calculated the outcome effect index, here meaning the effect of an outcome received during either exploration or a rule on future choices (see Methods). In each monkey, we found that outcomes received during exploration had a smaller effect on the next choice (**Fig 4E**; both monkeys, mean change in the 1-trial outcome effect index 0.63, 95% CI = [0.59, 0.68], p < 0.0001, t(88) = 28.2: monkey B = 0.62, 95% CI = [0.54, 0.71], p < 0.0001, t(26) = 14.9; monkey C = 0.63, 95% CI = [0.58, 0.69], p < 0.0001, t(61) = 23.8). Monkeys also learned more quickly about outcomes experienced during exploration, as indexed by a greater rate of decay in the influence of these outcomes (model fits illustrated in **Fig 4E**; both monkeys: explore learning rate = 1.52, 95% CI = [1.30, 1.73], rule learning rate = 0.80, 95% CI = [0.69, 0.92]; other explore parameters: scale = 4.81, 95% CI = [3.81, 5.80], offset = 0.65, 95% CI = [0.62, 0.68]; other rule paramters scale = 1.31, 95% CI = [1.81, 1.44], offset = 0.49, 95% CI = [0.46, 0.51]; monkey B, explore learning rate = 1.20, 95% CI = [0.93, 1.47], rule learning rate = 0.38, 95% CI = [0.32, 0.44); monkey C, explore learning rate = 1.73, 95% CI = [1.42, 2.04], rule learning rate = 0.87, 95% CI = [0.74, 1.01]). Thus, the model labeled as exploratory choices were reward-maximizing choices in which learning was enhanced.

### Cocaine reduces HMM-inferred exploration

**Fig 5:**
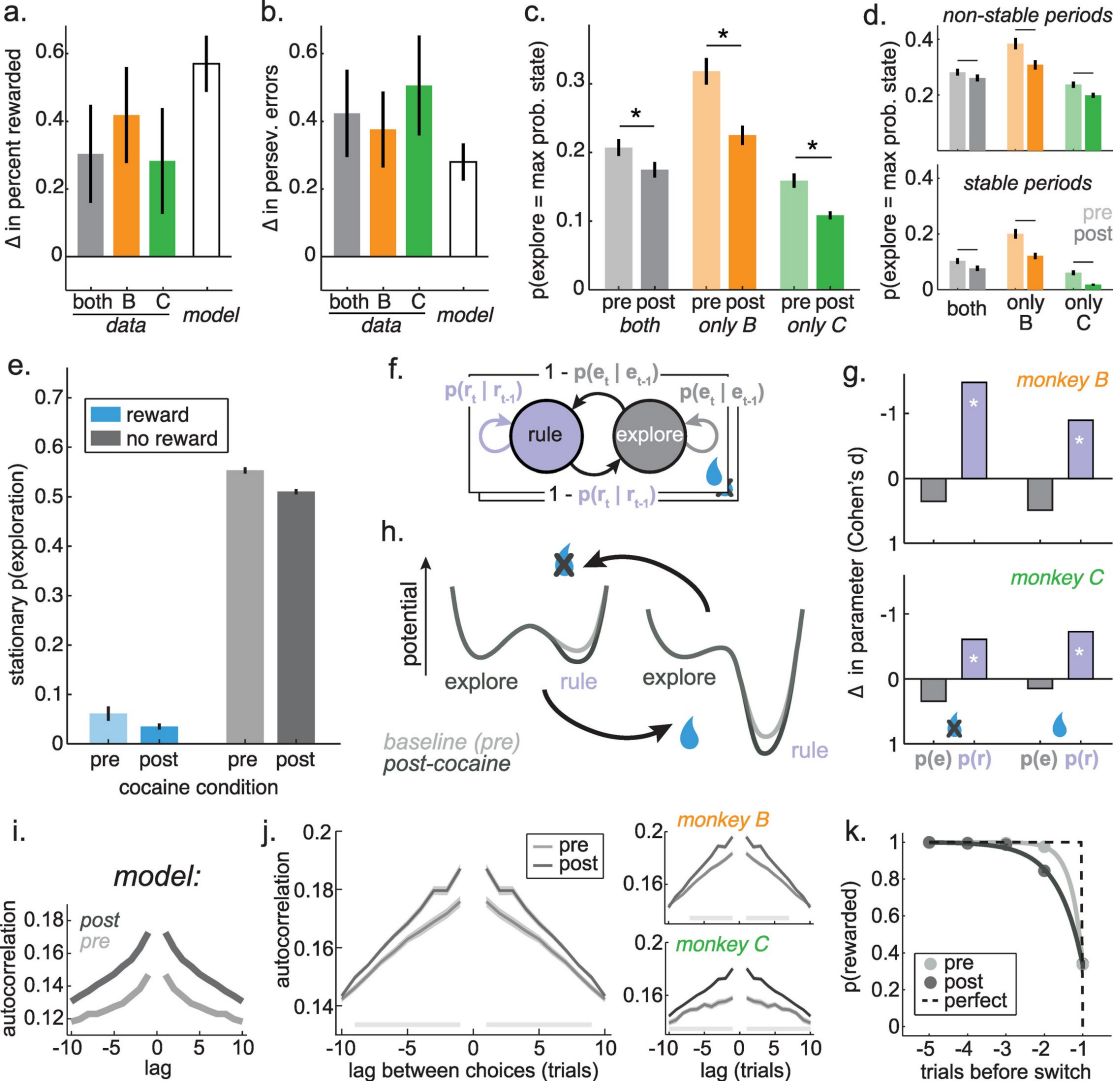
HMM predictions and effects of cocaine on model behavior. A) The increase in the probability correct after cocaine. Plotted separately for both monkeys together (gray bar), monkey B (orange) and monkey C (green), next to the increase in probability correct in simulated data from the model (white bar). Bars: Satterthwaite approximation of the +/ 99 CI. B) Same as A, for change in perseverative errors. C) The probability that exploration was identified as the most probable cause of each choice, before and after cocaine. Gray = both monkeys together, orange = monkey B, green = monkey C. Bars +/- SEM. D) Same as C, but with explore choices separated according to whether these occurred during stable periods (10 trials before change point; bottom) or elsewhere in the task (top). E) The stationary probability of the explore state, given the outcome of the previous trial (rewarded = blue, not rewarded = gray) and the cocaine condition (pre = before cocaine, post = after). F) Illustration of the 2 free parameters in each plate of the model (4 parameters total). E) Effect of cocaine on the model parameters. Change in parameters (Cohen’s d, post-cocaine minus baseline) in monkey B (top) and monkey C (bottom). * p < 0.05, t-test (see Table 1). Note that the slight decrease in the probability of staying in exploration was likely due to practice (see Results). H) A cartoon illustrating the effect of cocaine on model parameters (see Table 1) in terms of an attractor landscape. Here, exploration and rule adherence correspond to some local minima in a behavioral landscape, across which the monkeys move stochastically. Reward outcomes act to shift the baseline landscape (light line) from strongly favoring rule adherence following reward delivery (left) to a slight preference for exploration following reward omission (right; compare to panel D). Cocaine (dark line) globally increases the duration of rule-states, which suggests that it specifically deepens the attractor basin corresponding to rules, regardless of reward outcome. I) The autocorrelations of neighboring choices in data simulated from the model fit to pre- and post-cocaine data. J) The same choice autocorrelations in both monkeys (left), as well as in each monkey individually (right). Ribbons = SEM. Shaded lines = bins with significant offset between pre- and post-cocaine sessions, Holm-Bonferroni corrected for multiple comparisons. K) The reward history kernel preceding switches away from repeated choices to the same option (min. 5 trials) before and after cocaine administration. Error bars = SEM and are smaller than the size of the symbols.

First, we asked whether the model was capable of reproducing the major behavioral effects of cocaine. We fit one model to all the baseline sessions and a second model to the post-cocaine sessions, then simulated observations from each model. The changes in model parameters across the baseline and post-cocaine sessions were sufficient to reproduce the major behavioral results: an increase in both task performance (**Fig 5A**; mean increase in percent correct = 14.5%, 95% CI = 12.8 to 16.1%, p < 0.0001, t(145) = 17.70) and perseverative errors (**Fig 5B**; mean increase in percent perseverative errors = 4.8%, 95% CI = 3.9 to 5.8%, p < 0.0001, t(145) = 9.89). Thus, this descriptive model captured the main effects of cocaine on behavior.

**Table 1:** Effects of cocaine on model parameters. Mean parameter estimate (standard deviation) across all models. p(e_t_) = probability of exploration. p(r_t_) = probability of rule. Bold: significant change in post-cocaine sessions, relative to baseline within each monkey: * p < 0.05, ** p < 0.005, *** p < 0.0001, t-test (see Results for test statistics).

Parameter		Monkey B		Monkey C	
		Baseline	Post-cocaine	Baseline	Post-cocaine
**Reward**	**p(r**_**t**_**|r**_**t-1**_**)**	0.978 (0.008)	0.984 (0.006)**	0.995 (0.005)	0.998 (0.002)**
	**p(e**_**t**_**|e**_**t-1**_**)**	0.73 (0.17)	0.64 (0.21)	0.30 (0.30)	0.25 (0.25)
**No reward**	**p(r**_**t**_**|r**_**t-1**_**)**	0.02 (0.07)	0.19 (0.14)***	0.04 (0.11)	0.11 (0.12)*
	**p(e**_**t**_**|e**_**t-1**_**)**	0.28 (0.16)	0.22 (0.17)	0.18 (0.14)	0.14 (0.12)

Next, we asked whether cocaine affected the probability of exploration, as inferred from the model (see Methods). The monkeys had different levels of exploration, but within each monkey, there were fewer explore-state choices in post-cocaine treatment sessions, compared to baseline sessions (**Fig 5C**; monkey B: p < 0.0002, t(58) = 4.03, mean change = -9.3%, 95% CI = -4.7 to -13.9%; monkey C: p < 0.004, t(85) = 3.01, mean = -5.0%, 95% CI = -1.7 to -8.4%; not due to practice: β_1_ = 0.052, p < 0.03; β_2_ = 0.011, p = 0.3). Thus, monkeys explored less often after cocaine delivery, consistent with the idea that cocaine alters tonic exploration.

This effect was not driven by a change in the probability of exploration during specific epochs of the task. Instead, cocaine decreased the probability of exploration during both the stable periods (**Fig 5D:** 10 trials before change points: monkey B: p < 0.0002, t(58) = 4.03, mean change = -8.0%, 95% CI = -4.0 to -11.9%; monkey C: p < 0.002, t(85) = 3.20, mean = -4.3%, 95% CI = -1.6 to -7.0%) and during all other periods of the task, excluding these stable periods (monkey B: p < 0.005, t(58) = 3.02, mean change = -7.6%, 95% CI = -2.6 to -12.7%; monkey C: p < 0.05, t(85) = 2.07, mean = -3.8%, 95% CI = -0.1 to -7.5%). Thus, cocaine decreased the probability of exploration, regardless of whether that exploration was occurring when it was helpful or when it was not.

### Effects of cocaine on model dynamics

The stationary distribution of a HMM is the equilibrium probability distribution over states [53]. Here, this means the relative occupancy of explore-states and rule-states that we would expect after infinite realizations of the model's dynamics, given the outcome of the last trial (see Methods). The stationary distribution of the model thus provides a measure of the energetic landscape of the behavior the model is fit to. If a state has very low potential energy—if it is very sticky and its basin of attraction is deep—then we will be more likely to observe the process in this state, and the stationary distribution will be shifted towards this state [54]. Therefore, we will refer to the stationary distribution probability of exploration as the “relative depth” of exploration.

As expected, reward delivery reduced the relative depth of explore states (increased the relative depth of the rule states: **Fig 5D;** see Methods; β_1_ = -0.49, p < 0.0002). Cocaine also decreased the relative depth of explore states (β_2_ = -0.05, p < 0.02). There was a significant offset between monkeys (β_4_ = -0.05, p < 0.0002) and no effect of practice (β_5_ = 0.0003, p = 0.4) or interaction between reward and cocaine (β_3_ = 0.016, p = 0.4). This suggested that cocaine uniformly altered the depth of exploration, rather than the effect of reward on exploration. To test this, we asked whether the effect of cocaine on explore state depth differed after reward delivery, compared to reward omission. There was no significant difference after controlling for the expected effect of differing baselines (see Methods; paired t-test: p = 0.9, t(144) = -0.09, mean change = 1%, 95% CI = -25% to 23%). Moreover, the depth of exploration was correlated across reward outcome within the baseline sessions (both monkeys: r = 0.38, p < 0.0001, n = 89) and cocaine delivery did not disrupt these correlations (both monkeys: Pearson’s r = 0.23, p < 0.005, n = 147). Thus, cocaine uniformly decreased the relative depth of exploration, regardless of reward outcomes.

### Effects of cocaine on model parameters

Did cocaine reduce the relative depth of exploration by decreasing the depth of exploration or by increasing the depth of rule states? To arbitrate between these interpretations, we next asked how cocaine changed the parameters of the model. The model had 4 parameters (**Fig 5E**), reflecting the probability of staying in each of the two states (explore and the generic rule state) following the two outcomes (reward delivery and omission). If cocaine largely affected the probability of staying in exploration, then that would suggest that cocaine specifically decreased the depth of explore states. This is because the average dwell time in a state (that is, the inverse of the rate of leaving that state) has a natural relationship to the energetic depth of that state, relative to the energy barrier between states [55]. Alternatively, if cocaine largely affected the probability of staying in a rule, then that would suggest that cocaine specifically increased the depth of rule states. We also considered a third possibility: that cocaine had different effects following reward delivery and omission—i.e. decreasing the depth of rules after reward omission, but increasing depth of exploring after reward delivery. This last effect would be hard to reconcile with the idea of a unified effect on tonic exploration.

Within each monkey, there were significant changes in the same two model parameters in post-cocaine sessions (**Table 1**). Cocaine increased the probability of staying in rule states following reward omission (monkey B: p < 0.0001, t(58) = 5.69; monkey C: p < 0.02, t(85) = 2.57; not due to practice: β_1_ = 0.070, p < 0.04, β_2_ = 0.027, p = 0.1) and cocaine increased the probability of staying in rule states following reward delivery (monkey B: p < 0.001, t(58) = 3.45; monkey C: p < 0.003, t(85) = 3.06; not due to practice: β_1_ = 0.004, p < 0.01, β_2_ = 0.0002, p = 0.8). Cocaine had no significant effect on the depth of explore states following either reward omission (β_1_ = -0.004, p > 0.9) or reward delivery (β_1_ = 0.03, p = 0.7). However, there was a trend towards a decrease in the depth of explore states with practice in both conditions (omission: β_2_ = -0.03, p = 0.1, delivery: β_2_ = -0.06, p = 0.09), which could indicate more efficient patterns of exploration with experience in the task. Nevertheless, the weight of evidence suggests that cocaine selectively deepened rule states (Fig 5E): it decreased tonic exploration via increasing the tendency to adhere to a rule, regardless of reward outcomes.

### Effects of cocaine on the momentum of decision-making

Deepening rule-state attractor basins would make rules more stable across trials, meaning choices would be less likely to change, but not because animals are learning more slowly. Instead, deepening rule-states would increase choice momentum: the tendency of a choice policy, once established, to persist, regardless of any external influences. If cocaine increased choice momentum here, then we would see specific evidence of this in behavior, such as an increase in the autocorrelation length of choices and that a larger perturbation is required to change established choice patterns.

Indeed, choice autocorrelations were increased in model-simulated data after cocaine administration (**Fig 5I)**. Similarly, within the data, we found that nearby choices were more strongly autocorrelated in the post-cocaine sessions than the pre-cocaine sessions (**Fig 5J**; see **Methods**; sig. increase in autocorrelations at trial lags 1 through 9 in both monkeys, p < 0.05, Holm-Bonferroni correction for multiple comparisons). This suggests that cocaine increased the extent to which choices depended on previous choices. To determine whether the monkeys’ choices were less perturbable, we estimated how much external evidence was required to change behavior before and after cocaine by examining switch-triggered reward history. Pre-cocaine, the monkeys had close to the optimal reward history kernel shape, though they did occasionally switch even without experiencing a reward omission (**Fig 5K**). However, after cocaine exposure, the monkeys reward history kernels elongated: more evidence was required to switch (see **Methods**; the optimal decay parameter would approach 0, mean decay pre-cocaine: 0.27 ± 0.33 STD, mean decay post-cocaine: 0.66 ± 0.24 STD, difference in means = 0.39, 95% CI = 0.29 to 0.49, sig. difference, p < 0.0001, t(145) = 7.75; no significant change in the weight of the last outcome, p > 0.8). Thus, cocaine increased the momentum of choices.

## Discussion

These results suggest that the same process that facilitates flexibility in a dynamic environment is responsible for at least some spontaneous lapses in rule adherence when the environment is stable. This conclusion is based on the observation that spontaneous lapses and perseverative errors are not independent observations. They inversely co-varied across monkeys and sessions, but not because of failures to learn the reward contingencies in any given block. Instead, there was a common, tonic cause—both effects were the result of some latent process which caused deviations from established decison policies, both when these deviations were useful and when they were not. Moreover, lapses in this task were not simple errors, but instead information-maximizing choices, in which learning was enhanced, that occurred most frequently early in the task—when exploration was most valuable, but fatigue was minimal. We were able to perturb this process with chronic cocaine exposure. This perturbation is known to decrease flexibility by increasing perseveration [22–26,31], but here it actually improved performance in a set-shifting task by adjusting the shared process underlying both lapses and perseverative errors.

Together, these results suggest that exploration occurs tonically. This stands in contrast to phasic views of exploration that posit that exploration occurs nearly exclusively at the moments when it is most useful to the animal (e.g. [13,18,43]). Our use of the terms “tonic” and “phasic” here are novel. They are inspired by terms used to describe different patterns of locus coerrulus activity: the tonic pattern, where neurons are active continuously, without respect to task events, versus the phasic pattern, where LC activity is locked to surprising events or important task information [56]. It is important to note that these terms refer to different hypotheses about when exploration occurs, rather than what is explored. Here, exploration occurred tonically, but was still directed, rather than random—meaning that it targeted information-maximizing options [12].

To delineate precisely how cocaine altered tonic exploration, we turned to model-based analyses of the dynamics of behavior. We rerepresented choice patterns during each session in the transition matrices of a hidden Markov model. Analyzing these matrices allowed us to examine the energetic landscape of behavior. Here, we found that the effects of cocaine could be parsimoniously described as deepening attractor basins corresponding to rule states—cocaine essentially stabilized behavioral policies. As decision-making unfolds over many trials, deepening these attractor basins would increase the momentum of decision-making—meaning that a choice policy, once established, will persist for longer and require larger perturbations to change.

There is precedent for the idea that behavioral policies have momentum, both from experimental [57,58] and normative [59] perspectives. In decision-making, the term “choice hysteresis” is used to describe the common observation that subjects tend to repeat their previous choices more than reinforcement learning (RL) and other reward inference models naturally predict [8,57,58]. Indeed, many common extensions to RL models increase the models’ capacity to account for choice momentum [60,61], even when these extensions are described as implementing other psychological processes such as forgetting [62–64] or optimism [65]. Moreover, adding choice hysteresis to an RL model improves model fit to cocaine-treated animals, at least some of whom exhibit both increased choice hysteresis and decreased decision noise [66]—reinforcing our conclusion that cocaine exposure increases choice momentum. Additional work is necessary to determine how cocaine’s effects can be explained though a RL framework, ideally though comparing both a basic Q-learning model and a variety of the extensions known to account for choice momentum.

Here, we found that choice momentum was not introduced by cocaine, but instead it is an natural force in behavior that is upregulated by cocaine. From a normative perspective, choice momentum could be due to an evolutionary adaptation to the typical statistics of natural environments, which are often strongly autocorrelated [59]—a situation where momentum can facilitate learning [67]. This is because momentum ensures that decision-makers integrate information over multiple samples before changing their behavior—essentially filtering out noise [68]. Of course, it is possible that other interventions might regulate the likelihood of exploration through mechanisms unrelated to choice momentum, perhaps by regulating noise in action selection [8,12,69,70] or value learning [71]. However, it is possible that these mechanisms, like optimism and forgetting, are not altogether distinct from changes in choice momentum. Future work is necessary to determine whether these models can be reconciled within a common framework, perhaps by examining how changes in the parameters of mechanistic models affect the dynamics of decision-making.

### Relationship to theories of lapses and flexibility

We found that at least some lapses of task performance are due to the same exploratory mechanisms that allow us to adapt to a changing environment. However, we are not proposing that tonic exploratory noise is categorically different from other views of lapses, which cast these as the result of memory deficits, sensorimotor noise, or attentional or executive disengagement [3–6]. Instead, our view is that some of these constructs may be valid psychological descriptions of the effect that exploratory noise has on behavior.

In the brain, exploratory noise seems to produce effects that are consistent with a disruption in prefrontal control. For example, exploratory decisions are associated with sudden disruption in the functional organization of populations of neurons the prefrontal cortex [8,43]. It is possible that this disorganization reflects a disruption of the prefrontal dynamics underlying temporally extended cortical states such as working memory [72–76], motor control [77], decision-making [78–80], and executive control [81,82]. However, disrupting prefrontal control does not necessary imply disengagement. For example, reward-dependent learning is actually enhanced in the midst of this disruption in prefrontal organization [8] and disengaging the prefrontal cortex could allow behavior to be more tightly coupled to the environment [83]. Thus, disrupting prefrontal control may permit discovery via selectively randomize behavior with respect to information or policies held in the prefrontal cortex without causing disengagement per se.

On the surface, the link between lapses and perseverative errors that we report here may appear to conflict with previous views of errors in similar tasks as reflecting dissociable cognitive processes. Many modern theories of flexibility view perseveration as measuring the inability to inhibit a previous rule and lapses as measuring the inability to either maintain a rule or to inhibit distraction from irrelevant options [17,84–88]. The present results can be reconciled with these theories if increasing momentum of a rule makes the rule both easier to maintain over time and harder for distractors to compete with in the moment. Changing the momentum of a rule could decrease distraction simply by regulating the frequency of exploration, but it could also decrease distraction by regulating the strength of rule-relevant processes. Certainly, there is some evidence internal states linked to exploration [89] also predict increased distraction [90,91]. Future work—ideally combining cocaine administration with chronic population recording—is necessary to determine whether the momentum of rules is determined by changes in the strength of rule-related processing in the brain.

### Relationship to previous views of cocaine

The fact that cocaine administration increases perseveration is well-established [22–26,31]. However, here, cocaine also paradoxically improved overall performance in a set-shifting task—the exact type of task in which perseveration should make performance worse, not better. Our observation that cocaine can improve performance in a set-shifting task does have precedent in the literature. At least one previous study reported that cocaine addicts perform better than controls in the Wisconsin Card Sorting Task [35]. Our results suggest that this previous study was not an anomaly. Instead, in a within-subjects, causal study, we find that chronic cocaine administration is sufficient to both increase perseveration and improves set-shifting task performance. Further, we have proposed that these seemingly contradictory results can be reconciled if chronic cocaine administration decreases tonic exploratory noise. In our view, cocaine increases perseveration when the environment changes, but also makes it harder to spontaneously break from using a rule—because these are opposing sides of the same underlying process.

The perseverative effects of chronic cocaine use have previously been interpreted as a shift from goal-directed, action-outcome, or model-based control systems to habitual, stimulus-response, or model-free control systems [22–26,92–94]. Our results are partially compatible with these views because cocaine did make decisions more habitual—learning was slowed and choices changed more slowly over time. However, this did not occur at a stimulus-response level, but rather at the level of the the latent goals underlying choices. Thus, if anything, cocaine made subjects more habitual in their use of a model. This may seem like a contradiction in terms, but it is important to note that the habit/goal-directed dichotomy does not always map cleanly onto the model-based/model-free framework [95]. We are certainly not the first to note the link between exploratory noise and model-free/model-based decision-making [14], but more work is needed to understand how exploration interacts with model-based decision-making.

Our conclusion that cocaine increases the momentum of established policies is consistent with previous observations that cocaine selectively interferes with learning when a previously-learned response must be overcome [24,25,31] and observations that cocaine directly increases the probability of repeating responses [93,96]. However, it is important to note that our view is not that cocaine increases repetition at the level of choice, but instead that it increases the momentum of latent states underlying autocorrelations in choice.

### Basic insights into flexibility

The lawful relationship we find between lapses and perseverative errors was not an artificial consequence of cocaine exposure. Instead, cocaine shifted behavior along the axis of endogneous co-variability that already existed between these error types. Tonic exploration was a meaningful parameter that was controlled by cocaine administration, not introduced by it. Thus, the neurobiological targets of cocaine exposure may be promising targets for understanding the neural basis of tonic exploration.

One important cortical target of chronic cocaine administration is the orbitofrontal cortex (OFC) [25,32,97]: a region that is implicated in rule encoding [2,38,98–100]. Orbitofrontal damage leads to a deficit in maintaining performance during stable, steady periods in the WCST [101] and results in choice behavior that is consistent with an inability to learn or maintain rules ([102]; though see [103]). Of course, other cortical regions are also likely to contribute to regulating flexibility, particularly the anterior cingulate cortex [90,104], and there are functional and structural difference in both the cingulate and the OFC in chronic cocaine exposure [98,105]. Thus, these region are an important target for future studies of both cognitive flexibility and the effects of drugs of abuse.

Cocaine exposure also has profound effects on the brains’ neuromodulatory landscape. Chronic cocaine alters the dopamine (DA) [106–110], norepineprine (NE) [107,111,112], acetylcholine (ACh) [108,109], and serotonin [107] systems. ACh, DA and NE, in particular, have been previously implicated in regulating exploratory decision-making [56,113,114]. Moreover, lesions of ACh interneurons in the dorsomedial striatum may be sufficient to produce a change in lapse rates and perseverative errors simular to those reported here [115]. Thus, the effects of cocaine here support hypotheses linking these neuromodulatory systems to exploration.

## Conclusions

Why would we explore even when it has no strategic benefit? One possibility is that tonic exploration may have conferred such substantial benefits over evolutionary time that our brains evolved to continue to explore it even when it has no value in the moment. With tonic exploration, there is no need to calculate the value of exploration at each time step, which could reduce the energetic and/or computational costs of deciding when to explore [14]. Moreover, in natural environments, tonic exploratory noise could provide a valuable “interrupt” on temporally extended goal states, allowing organisms to occasional search for biologically important stimuli, such as predators or prey [116]. Although these results cannot nail down a single definitive evolutionary explanation, they provide support for the idea that considering evolutionary factors can help us to understand decision-making in a wide variety of tasks [117–119].

## Methods

### General surgical procedures

All animal procedures were approved by the University Committee on Animal Resources at the University of Rochester and were conducted in accordance with the Public Health Service’s Guide for the Care and Use of Animals. Two male rhesus macaques (Macaca mulatta) served as subjects. The animals had previously been implanted with small prosthetics for holding the head (Christ Instruments), which allowed us to monitor eye position and use this as the response modality. These procedures have been described previously [120]. To allow for chronic cocaine self-administration, we also implanted a subcutaneous vascular access port (VAP) in these animals (Access Technologies, Skokie, IL, USA), which was connected via an internal catheter to the femoral vein. Additional details of the VAP implantation procedure have been reported previously [106,121]. The VAP allowed monkeys to self-administer cocaine daily, and obviated the need for chemical or physical restraint, which might have unintended consequences for behavior. Animals received appropriate analgesics and antibiotics after all procedures, per direction of University of Rochester veterinarians. The animals were habituated to laboratory conditions and trained to perform oculomotor tasks for liquid reward before training on the conceptual set shifting task (CCST) began. Both animals participated in laboratory tasks for at least two years before the present experiment. Subjects had never previously performed a task-switching paradigm before training with this task. Previous training history for these subjects included two types of gambling tasks [120,122], two simple choice tasks [123,124], and a foraging task [125].

### Self-administration protocol

The monkeys sat in a primate chair placed in a behavioral chamber with a touchscreen (ELO Touch Systems, Menlo Park, CA, USA). Syringe Pump Pro software (Version 1.6, Gawler, South Australia) controlled and monitored a syringe pump (Cole Parmer, Vernon Hills, IL, USA), which delivered cocaine into the monkeys’ VAP. Monkeys pressed a centrally located visual cue on the touchscreen to obtain venous cocaine injections (cocaine provided by National Institutes of Drug Abuse, Bethesda, MD, USA), delivered in a 5 mg/ml solution at a rate of 0.15 ml/s. Monkeys were acclimated to cocaine self-administration across ten days of training, during which the response requirement and dose increased from 3 responses/reward (FR3) and 0.1 mg/kg (0.8 mg/kg of cocaine daily) to 30 responses/reward (FR30) and 0.5 mg/kg (4 mg/kg of cocaine daily). Monkeys were given 3 hours to complete infusions each day (in practice, monkeys typically completed the all 8 infusions within 1–2 hours). Monkeys self-administered cocaine 5 days a week.

### Behavioral task

Specific details of this task have been reported previously [37–40]. Briefly, the present task was a version of the CSST: an analogue of the WCST that was developed for use in nonhuman primates [36]. Task stimuli are similar to those used in the human WCST, with two dimensions (color and shape) and six specific rules (three shapes: circle, star, and triangle; three colors: cyan, magenta, and yellow; Fig 1A). Choosing a stimulus that matches the currently rewarded rule (i.e. any blue shape when the rule is blue; any color of star when the rule is star) results visual feedback indicating that the choice is correct (a green outline around the chosen stimulus) and, after a 500 ms delay, a juice reward. Choosing a stimulus that does not match the current rule results in visual feedback indicating that the choice is incorrect (a red outline), and no reward is delivered after the 500 ms delay.

The rewarded rule was fixed for each block of trials. At the start of each block, the rewarded rule was drawn randomly. Blocks lasted until monkeys achieved 15 correct responses that matched the current rule. This meant that blocks lasted for a variable number of total trials (average = 22.5), determined by both how long it took monkeys to discover the correct objective rule and how effectively monkeys exploited the correct rule, once discovered. Block changes were uncued, although reward-omission for a previously rewarded option provided noiseless information that the reward contingencies had changed.

On each trial, three stimuli were presented asynchronously, with each stimulus presented at the top, bottom left, or bottom right of the screen. The color, shape, position, and order of stimuli were randomized. Stimuli were presented for 400 msec and were followed by a 600-msec blank period. (The blank period was omitted from Fig 1A because of space constraints). Monkeys were free to look at the stimuli as they appeared, and, though they were not required to do so, they typically did [37]. After the third stimulus presentation and blank period, all three stimuli reappeared simultaneously with an equidistant central fixation spot. When they were ready to make a decision, monkeys were required to fixate on the central spot for 100 msec and then indicate their choice by shifting gaze to one stimulus and maintaining fixation on it for 250 msec. If the monkeys broke fixation within 250 milliseconds, they could either again fixate the same option or could change their mind and choose a different option (although they seldom did so). Thus, the task allowed the monkeys ample time to deliberate over their options, come to a choice, and even change their mind, without penalty of error.

### Data analysis

Data were analyzed with custom MATLAB scripts and functions. All t-tests were two-sample, two-sided tests, unless otherwise noted. All generalized linear models (GLMs) included a dummy-coded term to account for a main effect of monkey identity (1 for monkey B, 0 for monkey C) and were fit to session-averages, rather than individual trials. One session (1/147) was excluded from these analyses because one of its transmission matrices did not admit a stationary distribution. No data points were excluded for any other reason. Observation counts for each analysis are reported in figure legends and/or Results.

#### Operational definitions of errors

Lapses were defined as errors that occurred during stable periods (the 10 trials immediately preceding change points) and deviated from the previous choice’s color or shape. Lapse-like errors were defined as errors that deviated from the previous choice’s color or shape, but unlike lapses, lapse-like errors could occur anywhere in the session. Perseverative errors were defined as errors that occurred in the period of maximal learning (5 trials following change points) that did not deviate from the previous choice’s color or shape. The number of errors varied widely in frequency across sessions due to differences in how much the monkeys used a random versus directed strategy for exploration (errors occurred on 22% to 48% of the 5 post-change-point trials), so perseverative error frequency was normalized to the total number of error trials in the post-change-point epoch.

#### Outcome effect index

We quantify the amount of learning within session or from specific past rewards with the “outcome effect index”. This is essentially a measure of how much some past reward outcome received on some past trial τ influences the probability of repeating either of the past choice’s features on the current trial. We calculate this as:

$$\frac{p(repeat_{t}|reward_{t-\tau }=1)-p(repeat_{t}|reward_{t-\tau }=0)}{p(repeat_{t})}$$

Where normalizing by p(repeat) controls for different tendencies to repeat choices, irrespective of reward. This value is then averaged over color and shape to produce the outcome effect index reported in the text and figures.

To estimate the rate of learning from outcomes received during exploration and rules (**Fig 4E**), we predicted the outcome effect index for previous trials -1 through -5 using a 3-parameter decaying exponential function:

$$\text{outcome}\;{\text{effect}}_{t}=\text{offset}+\text{scale}\cdot e^{t\alpha }$$

Where the offset term captures an DC offset between the two conditions, the scale captures the outcome effect of the last trial (trial -1), and the alpha parameter captures the rate of decay in this influence over the following trials—that is, the learning rate.

#### Expected number of change 1 and change 2 lapses

To determine if monkeys were using directed exploration during lapses, we calculated the rate at which lapses would change from previous choices in either 1 or 2 dimensions by chance. There were two classes of last-trial/current-trial pairs. In the first, the identical choice from the last trial was available, so the only choices that could possibly lead to lapses were both change 2 dimension choices. In the second, the identical choice from the last trial was not available, so lapses would either target the non-rewarded feature from the last choice (changing 1 dimension) or target the other option (changing 2 dimensions). There were 3! = 6 possible permutations of color with respect to shape, 2 of which would be of the first type for any given choice, with a 0% chance of changing only 1 dimension, and 4 of which would be of the second, with a 50% chance of changing only 1 dimension. This gave us an expected probability of changing 1 dimension of (2/6) (0/2) + (4/6) (1/2) = 1/3 and an expected probability of changing 2 dimensions of (2/6) (2/2) + (4/6) (1/2) = 2/3. To account for this difference in chance levels, the counts of change 1 dimension and change 2 dimension errors were normalized by dividing by the expected count (product of the expected probability of lapse type and the total lapse count) in **Fig 2G**.

#### Identifying information-maximizing choices

To determine what choice(s) would maximize information about which feature was currently the best, we measured the information gain from different choice strategies in a model with a restricted, 1-trial memory. This was a reasonable approximation because the effects of previous choices and rewards tend to decay exponentially, meaning that the last trial is the one with the single biggest influence on choice. Assuming all possible pasts before the last choice at time t-1, we uniformly initialize the prior that each feature (f) is the best (f*) of the N_f_ features:

$$\hat{p}(f_{t-1}=f^{*})=\frac{1}{N_{f}}$$

After making the last choice at time t-1, we estimate the likelihood that the feature we chose was the best in a reward-dependent fashion. If the monkeys were rewarded:

$$p(f_{t-1}=f^{*})=\{\begin{array}{l} \frac{1}{2},\text{if}\;\text{choice}=\text{f}\\ 0,\text{otherwise} \end{array}$$

If the monkeys were not rewarded:

$$p(f_{t-1}=f^{*})=\{\begin{array}{l} 0,\text{if}\;\text{choice}=\text{f}\\ \frac{1}{N_{f}-2},\text{otherwise} \end{array}$$

Small amounts of noise, |N(0, 10^−4^)|, were added to all 0’s so that information would be computable. We then estimate which choice should be best on the current trial by multiplying the prior and posterior:

$$\hat{p}(f_{t}=f^{*})=\hat{p}(f_{t-1}=f^{*})p(f_{t-1}=f^{*})$$

Re-normalizing this to be a valid probability distribution gives a new prior about which stimulus feature is best going into trial t. To determine what choice at trial t would maximize information gain relative to this prior, we then simulate choices that differed in 0, 1, or 2 stimulus features from trial t-1, update the likelihood as we did previously, and then generate a new posterior estimate of which feature is best going forward:

$$\hat{p}(f_{t+1}=f^{*})=\hat{p}(f_{t}=f^{*})\hat{p}(f_{t}=f^{*})$$

We reasoned that the information-maximizing choice would be the one that caused the largest drop in uncertainty in this distribution. That is, it would be the choice, c, that maximizes:

$$\text{information}\;\text{gain}=H_{t}-H_{t+1}$$

The uncertainty about what feature was the best at time t is the prior entropy:

$$H_{t}=-\sum_{f}\hat{p}(f_{t}=f^{*})\log_{2}\hat{p}(f_{t}=f^{*})$$

Because there were two possible futures—one where the animal would be rewarded, and one where they were not and the likelihood of these futures depended on choice—we calculated the estimated future entropy as a weighted average of these possible futures:

$$H_{t+1}=-\sum_{f}\sum_{r\in 0,1}p(r|c=f)\hat{p}(f_{t+1}=f^{*}|r)\log_{2}\hat{p}(f_{t+1}=f^{*}|r)$$

Where we estimated the probability of reward for choosing each feature, p(r | c = f) by taking advantage of the fact that the probability that the monkeys would be rewarded for choosing a feature is proportional to the likelihood that this feature is the best. This means we can approximate the monkey’s internal estimate of reward probability from their prior on what feature is the best:

$$p(r|c=f)\propto \hat{p}(f_{t+1}=f^{*})$$

Supplemental Fig 2 illustrates both this estimated probability of reward and the the information gain for choices that differ in 0, 1, or 2 features.

#### Differentiating the effects of cocaine treatment from practice

Task performance reached stable levels in both monkeys before the baseline, pre-cocaine sessions began (Fig 2A). Nevertheless, we were concerned that putative effects of cocaine self-administration might instead be trivial consequences of the increased experience with the task in the post-cocaine sessions. Any effect of cocaine treatment would produce a step change in behavior that was aligned to the start of cocaine administration. Conversely, the effects of practice would change gradually across sessions. Therefore, to determine whether individual behavioral effects were due to practice or cocaine, we fit the following GLM to the session-averaged behaviors of interest:

$$\text{behavior}=\beta_{0}+\beta_{1}\cdot tx+\beta_{2}\cdot session+\beta_{3}\cdot monkey+\eta$$

Where “tx” is a logical vector indicating whether the session was conducted before or after chronic cocaine self-administration (a step change term) and “session” was a vector of session number within the experiment for each monkey (a gradual ramping term). One additional term “monkey” accounted for the random effect of monkey identity, and the model included the standard intercept and noise terms (β_0_ and η, respectively). Thus, β_1_ captured any offset due to chronic cocaine administration, while β_2_ captured any effect of practice for each analysis.

#### Probability of novel choices

Only 3 of the 9 possible stimuli (i.e. 9 combinations of 3 colors and 3 shapes) were available on each trial, so the likelihood of repeating choices that shared neither feature was constrained by the available options. Therefore, we calculated the monkeys’ probability of choosing each number of feature repeats as the total number of times a certain number of features was repeated, divided by how many times it was possible to repeat that number of features. Both terms were calculated within session.

#### Hidden Markov model

In the HMM framework, choices (y) are “emissions” that are generated by an unobserved decision process that is in some latent, hidden state (z). Latent states are defined by both the probability of each emission, given that the process is in that state, and by the probability of transitioning to or from each state to every other state. Straightforward extensions of this framework allow inputs, such as rewards, to influence state transitions [52], in which case the latent states can be thought of as a discretized value function.

The observation model for each hidden state is the probability choosing each option when the process is in that state. These emissions models differed across the two broad classes of states in the model—the explore states and rule states—based on the fact that there were two different dynamics in the choice behavior: one reflecting random choosing while exploring and one reflecting long staying durations due to persistent rules (S1 and S2 Figs). Therefore, the observation model for any choice option n during explore states was:

$$p(y_{t}=n|z_{t}=\text{explore})=\frac{1}{N}$$

Where N is the number of stimuli that were presented (i.e. N = 3). During rules, the observation model was conditioned on whether or not each stimulus is in the current rule set:

$$\begin{array}{l} p(y_{t}=n|z_{t}=rule_{i},n\in rule_{i})=1\\ p(y_{t}=n|z_{t}=rule_{i},n\;\notin \;rule_{i})=0 \end{array}$$

The latent states in this model are Markovian meaning that they are time-independent. They depend only on the most recent state (z_t_) and most recent reward outcome (u_t_):

$$P(z_{t}|z_{t-1},u_{t-1},y_{t-1},\;\ldots \;,z_{1},u_{1},y_{1})=P(z_{t}|z_{t-1},u_{t-1})$$

This means that the probabilities of each state transition are described by reward-dependent transmission matrix, A_k_ = {a_i,j_}_k_ = P(z_t_ = j | z_t-1_ = i, u_t-1_ = k) where k ϵ{rewarded, not rewarded}. There were 7 possible states (6 rule states and 1 explore state) but parameters were tied across rule states such that each rule state had the same probability of beginning (from exploring) and of sustaining itself. Similarly, transitions out of explore were tied across rules, meaning that it was equally likely to start using any of the 6 rules after exploring. Because monkeys could not divine the new rule following a change point and instead had to explore to discover it, transitions between different rule states were not permitted. The model assumed that monkeys had to pass through explore in order to start using a new rule, even if only for a single trial. Thus, each plate k of the transition matrix had only two parameters, meaning there were a total of 4 parameters in the reward-dependent model.

The model was fit via expectation-maximization using the Baum Welch algorithm [53,126]. This algorithm finds a (possibly local) maxima of the complete-data likelihood, which is based on the joint probability of the hidden state sequence Z and the sequence of observed choices Y, given the observed rewards U:

ℒ(Θ|Y,Z,U)=P(Z,Y|U,Θ)

The complete set of parameters Θ includes the observation and transmission models, discussed already, as well as an initial distribution over states, typically denoted as π. Because monkeys had no knowledge of the correct rule at the first trial of the session, we assumed the monkeys began in the explore state. The algorithm was reinitialized with random seeds 100 times, and the model that maximized the observed (incomplete) data log likelihood was ultimately taken as the best for each session. The model was fit to individual sessions, except to generate simulated data, in which case one model was fit to all baseline sessions and a second to all post-cocaine sessions. To decode latent states from choices, we used the Viterbi algorithm to discover the most probable a posteriori sequence of latent states [53].

To simulate data from the model, we created an environment that matched the monkeys’ task (choices between 3 options with 2 non-overlapping features and a randomly selected rewarded rule that changed after 15 correct trials). We then probabilistically drew latent states and choice emissions as the model interacted with the environment. The only modification to the model for simulation was that the choice of rule state following an explore state was constrained to match one of the two features of the last choice, chosen at random.

#### Stationary distribution

To gain insight into how cocaine changed the likelihood of rule states following reward delivery and omission, we examined the stationary distributions of the model. The transmission matrix of a HMM is a system of stochastic equations describing probabilistic transitions between each state. That is, each entry of a transmission matrix reflects the probability that the monkeys would move from one state (e.g. exploring) to another (e.g. using a rule) at each moment in time. In this HMM, there were two transmission matrices, one describing the dynamics after reward delivery and one describing the dynamics after reward omission. Moreover, because the parameters for all the rule states were tied, each transition matrix effectively had two states—an explore state and a generic rule-state that described the dynamics of all rule states. Each of these transition matrices (A_k_) describes how the entire system—an entire probability distribution over explore and rule states—would evolve from time point to time point given the outcome of the previous trial, k. You can observe how these dynamics would change any probability distribution over states π by applying the dynamics to this distribution:

$$\pi_{t+1}=\pi_{t}A_{k}$$

Over many iterations of these dynamics, ergodic systems will reach a point where the state distributions are unchanged by continued application of the transmission matrix as the distribution of states reaches its equilibrium. That is, in these systems, there exists a stationary distribution, π*, such that:

$$\pi^{*}=\pi^{*}A_{k}$$

If it exists, this distribution is a (normalized) left eigenvector of the transition matrix A_k_ with an eigenvalue of 1, so we solved for this eigenvector to determine the stationary distribution of each A_k_, if it had one. (Only one of the A_k_ matrices did not admit a stationary distribution, so this session was not included in analyses related to this measure.)

#### Analyzing stationary distributions

To determine how cocaine affected the relative depth of exploration and the generic rule state, we constructed a GLM. The model included terms to describe the effects of reward, cocaine, and the interaction between the two on the depth of exploration. This interaction allowed the model to describe a phasic, reward-dependent effect of cocaine on the depth of exploration, if it were present:

$$\begin{array}{l} depth=\beta_{0}+\beta_{1}(rwd)+\beta_{2}(cocaine)+\beta_{3}(rwd\times cocaine)+\ldots \\ \;\beta_{4}(monkey)+\beta_{5}(session) \end{array}$$

The model thus accounted for any offset between monkeys (“monkey”, 1 for monkey B, 0 for monkey C) or practice effects (“session”). It also included terms to describe the effects of reward (“rwd”, 1 for reward delivery, 0 for omission), cocaine (“cocaine”, 1 for pre-cocaine baseline sessions, 0 for post-cocaine sessions), and the interaction between reward and cocaine. This allowed the model to describe a phasic, reward-dependent effect of cocaine on model dynamics or a tonic, reward-independent form of exploration.

#### Comparing changes in probabilities

We calculated log odds ratios to compare the magnitude of changes in probability when baseline probabilities differed. Because probabilities are bounded, they are necessarily nonlinear transformations of an unbounded latent process of interest. This means that a fixed change in an underlying linear process can produce very different magnitude changes in probability, depending on the baselines. For intuition, picture a logistic function—a typical nonlinear transformation used to covert linear observations into probabilities. The effect of an equivalent change in the x-axis on the y-axis is depends on the baseline position on the x-axis: an identical shift on the x-axis has a large effect on y when x starts close to the midpoint of the function, but a small effect on y when x starts close to either end. The logit transformation linearizes the relationship between different observed probabilities because it is the inverse of the the logistic function:

$$logit(p)=logistic^{-1}=\log\left(\frac{p}{1-p}\right)$$

The difference between log odds (also known as the log odds ratio) then provides a linearized measure of effect magnitude that less sensitive to differing baseline levels. It is:

$$log(\text{odds}\;\text{ratio})=logit(p_{1})-logit(p_{2})$$

#### Choice autocorrelations

To measure the autocorrelations in choice in the real and model-simulated data, we coded each choice as 6-element indicator vector with each entry corresponding to a logical of whether the animal chose a particular stimulus feature (shape and color). We then calculated the choice autocorrelations independently for each stimulus feature (Pearson’s correlation). The average across the 6 stimulus features was taken as the mean choice autocorrelation.

#### Switch-triggered reward history kernel

To get a model-free estimate of how monkeys integrated past rewards when deciding to change their behavior, we calculated the effects of previous outcomes on switch decisions (decisions to change either color or shape) that occurred after a minimum of 5 decisions to the same color or shape (identical results with 10 decisions). We then fit a two-parameter decaying exponential curve:

$$weight=1-last\cdot e^{-x\cdot decay^{-1}}$$

Here, “last” corresponds to the probability that the animal was not rewarded on the last trial before a switch, and “decay” corresponds to the rate of decay in the switch triggered reward history kernel. Because reward omissions give perfect information that you should change your behavior in this context, the optimal decision maker would switch away from a good color or shape if and only if they were not rewarded on the last trial. This means that, provided the choice matched the rule and was reliably rewarding, the optimal decision maker would have last = 0 and decay approaching 0.

## Supporting information

S1 Fig Lapses do not anticipate change points. Because rule changes were triggered by 15 correct trials, it was possible that the monkeys anticipated rule changes by counting rewards. If this was the case, they might try to deviate from established policies in anticipation of the rule change, in order to avoid an inevitably incorrect trial. However, there was no increase in lapse rates during the stable epoch (the 10 trials before a rule change) in either the pre- or post-cocaine sessions (left). Similarly, neither lapses that differed in 1 or 2 dimensions from the previous choice anticipated the change points in the baseline sessions (right). Inset) Same, aligned post-change point. Here, the substantial increase in the rate of change 1 dimensional lapses on the first trial is due to the inevitable error—if subjects are following a stable policy on this trial, they will inevitably commit an error and will have a 50% chance of also changing 1 dimension from their previous choice (because the probability that this trial will only offer options that match the last choice in 1 dimension is 50%). Following this error feedback, the rate of change 2 dimensional lapses spikes, likely due to a self-avoiding, “smart” exploration strategy after change points.
(EPS)

S2 Fig Information gain and probability of reward for choosing stimuli that differ in 0, 1, or 2 dimensions from previous choices. Related to Figs 2G and 4D. Information gain and reward probability are plotted separately for trials following reward omission (left) and reward delivery (left). Following reward omission, the choice that maximizes both the probability of reward and information is the one that deviates in 2 stimulus dimensions from the last choice. Following reward delivery, the information-maximizing choice is the one that differs in 1 dimension, while the reward-maximizing choice would change 0 dimensions (repeating exactly the previous choice).
(EPS)

S3 Fig Hidden Markov Model development (related to Figs 4 and 5). To determine whether an HMM was an appropriate descriptive model for this dataset, we first asked whether there were different behavioral dynamics that might correspond to using a rule and exploring. One way to do this is to examine the distribution of runs of repeated choices within some choice dimension (Ebitz, Albarran, & Moore, 2018). If monkeys are exploiting a rule, then they would have to repeatedly choose options that are consistent with this rule. During a rule, runs of repeated choices—or interswitch intervals—would be long. However, exploration, monkeys need to briefly sample the options to determine whether or not they are currently rewarded. That is, during exploration runs of repeated choices should be very brief: on the order of single trials.To the extent that choice runs end stochastically (an assumption of the HMM framework), inter-switch intervals will be exponentially distributed. Moreover, if there are multiple latent regimes (such as exploring and rule-following), then we would expect to see inter-switch intervals distributed as a mixture of exponential distributions, because choice runs have a different probability of terminating in each latent regime. The distribution of inter-switch intervals (n interswitch intervals = 49,059) resembled an exponential (**left**), but was better described by a mixture of two discrete exponential distributions (blue lines; 1 exponential: 1 parameter, log-likelihood = -142077.0, AIC = 284156.1, AIC weight < 0.0001, BIC = 284165.6, BIC weight < 0.0001) than a single distribution (black line; 2 exponential: 3 parameters, log-likelihood = -119773.2, AIC = 239552.4, AIC weight = 1, BIC = 239580.7, BIC weight = 1). Adding additional exponential distributions did not improve model fit (**right**), suggesting that there were only two regimes (3 exponentials: 5 parameters, log-likelihood = -119773.2, AIC = 239556.4, AIC weight < 0.14, BIC = 239603.7, BIC weight < 0.0001; 4 exponentials: 7 parameters, log-likelihood = -119773.2, AIC = 239560.4, AIC weight < 0.02, BIC = 239626.6, BIC weight < 0.0001). The best-fitting model was thus the two-exponential mixture. It had one long-latency component (half life = 9.0), consistent with a persistent rule-following response mode. It also had one short latency component (half life 1.4), consistent with rapidly shifting between options.
(EPS)

S4 Fig Short choice runs occur more frequently than expected (related to Figs 4 and 5). Because rules only operated on either the color or shape of the option, we quantified the duration of inter-switch intervals independently within the color and shape domains (i.e. a magenta star choice followed by a magenta circle choice be counted as part of the same choice run in the color domain, but would part of different choice runs in the shape domains). This meant that choices would inevitably be randomized within one feature domain during repeated choices in the other domain. Thus, the existence of a mode with a short half-life is not sufficient evidence of short-latency search dynamics in this task. However, if randomization in the other domain was the sole cause of short duration samples, then observations from the short sampling mode would occur exactly as frequently as observations from the persistent mode. However, short choice runs occurred more frequently than expected. To determine this, we calculated the expected time in each state as the product of the average run length in that state and the probability of being in that state. Then, we normalized the expected time in the short state by the sum of expected times in all states. That is, this measure would be at 0.5 if observations from the short state were equally as frequent, and greater than 0.5 if they were more frequent. The expected number of short state observations was significantly greater than 0.5 (both subjects, paired t-test, p < 0.0001, t(88) = 17.02; subject B: p < 0.0003, t(26) = 4.18; subject C, p < 0.0001, t(61) = 27.6), indicating that both subjects had more frequent short duration samples than would be expected if those short duration samples were merely caused by choices along a different dimension. Thus, both subjects exhibited strong evidence for a separate search state, in which they made short duration runs of choices to the different options.
(EPS)

S5 Fig An input-output HMM accounts for reward-dependent decisions (related to Figs 4 and 5). Inter-switch intervals were largely exponential—consistent with the Markovian assumptions of an HMM—and we observed different search and rule dynamics. However, it is important to note that in the log plot (**top left**), there were significant deviations from the predictions of simple exponential mixture model. These were likely due to the changes in reward contingencies that were triggered each time 15 correct trials were completed. To account for this obvious dependence on reward, we extended a simple 2 parameter HMM model to allow state transition probabilities to depend on previous reward outcomes [52]. Accounting for this reward dependence (4-parameter ioHMM) reproduced these dynamics (**bottom left**) and improved model fit in both monkeys (**right**; both monkeys: 2 parameter HMM, log-likelihood = -39614, 4 parameter ioHMM, log-likelihood = -30240, log-likelihood ratio test: statistic 18749, p < 0.0001; monkey B: HMM, log-likelihood = -12973, ioHMM = -11714, log-likelihood ratio test: statistic = 2518.7 p < 0.0001; monkey C: HMM, log-likelihood = -26641, ioHMM = -18526, log-likelihood ratio test: statistic = 16230, p < 0.0001).
(EPS)

S6 Fig Model performance as a function of parameter combinations. To determine which parameter combinations for this model would be optimal in this task, we simulated the model’s performance for a variety of parameter combinations (15,000 simulated datasets of 100 sessions of 500 trials each under uniformly sampled parameters). Mean reward probability of reward for each parameter alone (left; +/- variance of a 10-degree polynomial curve fit) and for important pairs of parameters (right) are illustrated here. The optimal parameter combination is shown with an asterisk (*) on each graph and was calculated as the geometric mean parameters of the 7 sessions within 1% reward of the maximum reward probability that we simulated. This optimum corresponded to the parameters p(stay in rule | no reward) = 0.0436, p(stay in explore | no reward) = 0.5277, p(stay in rule | reward) = 0.9971, p(stay in explore | reward) = 0.0325. Note that performance increases monotonically with p(stay in rule | reward), the parameter that the lapse rate was most sensitive to (correlation between this parameter and lapse rate: -0.81).
(EPS)

S7 Fig Frequency of HMM-labeled states systematically differs at change points (related to Fig 4B). Right) Dark gray line indicates the probability that exploration was identified as the most probably state ± STD. The light gray line is the mean over sessions of label-shuffled data (100 permutations per session). Dots above the lines indicate bins where the unshuffled data was more likely to be explore-labeled than the shuffled data in greater than chance number of sessions (2.5%). Dots below the lines indicate bins where the unshuffled data was less likely to be explore-labeled in greater than chance number of sessions (2.5%). The real dataset was either more or less exploratory than the shuffled dataset in every bin, indicating that exploration was strongly structured with respect to the change points. Note that bin 14 is labeled as both greater and lower than chance. This is because exploration was above chance in a significant number of sessions and also below chance in a different significant number of sessions. Left) State probabilities for each individual session (dark gray) and label-shuffled data (light gray).
(EPS)
